# New Angucycline Glycosides from a Marine-Derived Bacterium *Streptomyces ardesiacus*

**DOI:** 10.3390/ijms232213779

**Published:** 2022-11-09

**Authors:** Cao Van Anh, Joo-Hee Kwon, Jong Soon Kang, Hwa-Sun Lee, Chang-Su Heo, Hee Jae Shin

**Affiliations:** 1 Marine Natural Products Chemistry Laboratory, Korea Institute of Ocean Science and Technology, 385 Haeyang-ro, Yeongdo-gu, Busan 49111, Korea; 2 Department of Marine Biotechnology, University of Science and Technology (UST), 217 Gajungro, Yuseong-gu, Daejeon 34113, Korea; 3 Laboratory Animal Resource Center, Korea Research Institute of Bioscience and Biotechnology, 30 Yeongudanjiro, Cheongju 28116, Korea

**Keywords:** *Streptomyces ardesiacus*, urdamycin, grincamycin, anti-bacterial, cytotoxicity

## Abstract

Chemical investigation of the ethyl acetate extract from the culture broth of the marine-derived actinobacterium *Streptomyces ardesiacus* 156VN-095 led to the isolation of three hitherto undescribed angucycline glycosides, including urdamycins W and X (**1** and **2**) and grincamycin U (**9**), as well as their seven known congeners. The structures of the new compounds were elucidated by means of spectroscopic methods (HRESIMS, 1D and 2 D NMR) and comparison of their experimental data with literature values. Compounds **1**–**3** and **9** were evaluated for their anti-Gram-positive bacterial effect and cytotoxicity against six cancer cell lines. Compound **1** displayed significant cytotoxicity against all the tested cell lines with GI_50_ values of 0.019–0.104 µM. Collectively, these findings highlight the potential of angucycline glycosides as leading structures for the development of new anti-cancer drugs.

## 1. Introduction

The genus *Streptomyces* are renowned as the largest contributor of currently used antibiotics [1]. Over the past decades, the most important classes of antimicrobial drugs have been isolated from this genus, such as tetracyclines, aminoglycosides, macrolides, and lypopeptides [2]. However, bioassay-guided screening of common actinomycetes, particularly the genus *Streptomyces*, often led to the rediscovery of known compounds and it is not an efficient approach to identifying new natural scaffolds [3]. Over the last decades, several dereplication strategies in natural product research were conducted to search for novel chemical structures from natural resources, such as bioactivity-guided fractionation, chemical profiles of crude extract collections and target compounds, and taxonomic identification of microbial strains [4]. Since terrestrial microbes have been well studied, several different approaches, including the investigation of unexploited habitats such as marine and extreme environments, have been employed to yield novel chemistry [5].

Polyketides are one of the largest classes of natural products isolated from the genus *Streptomyces* [6,7]. Among them, polycyclic aromatic polyketides, which are called “cytotoxic antibiotics”, are one of the chemically richest classes of secondary metabolites and possess various biological activities, predominantly anticancer and antibacterial, and some of them are currently used as anti-cancer or anti-bacterial drugs, such as doxorubicin, epirubicin, and tetracycline [8,9,10]. The angucycline group is the largest group of type II PKS-engineered natural products, and is abundant in biological activities and chemical scaffolds [8], and the majority of angucycline producers were classified to be *Streptomycetes* of various species [11]. Besides possessing an interesting cytotoxicity, some of the angucyclines act as hydroxylase and/or mono-oxygenase inhibitors, some potentially inhibit blood platelet aggregation and others demonstrate antibacterial or antiviral activity [11]. Therefore, angucyclines may serve as leading structures for new drug discovery.

As part of our ongoing studies on bioactive secondary metabolites isolated from marine-derived microorganisms, an actinomycetal strain was isolated near Nha Trang Bay, Vietnam, and identified as *Streptomyces ardesiacus* 156VN-095 by 16S rRNA gene sequence analysis. NMR and HPLC profiling of the ethyl acetate (EtOAc) extract from the culture broth of the strain showed characteristic signals of polycyclic aromatic polyketides. Chemical investigations of a large-scale culture of the strain were carried out and resulted in the isolation of three previously undescribed angucycline polyketides (**1**, **2**, and **9**) and their seven known congeners. Herein, we describe the isolation, structure elucidation, and their anti-Gram-positive bacterial and cytotoxic activities.

## 2. Results and Discussion

The strain *Streptomyces ardesiacus* 156VN-095 cultured on Bennett’s agar medium produced dark purple pigments. A small-culture test in Bennett’s modified broth led to the isolation of urdamycins A and B as major metabolites (Figure S1). To further discover other unknown chemical constituents, a large-scale culture was conducted, and consequently, three unreported congeners were isolated. The structures and biological activities of these compounds are described below.

**Figure 1:**
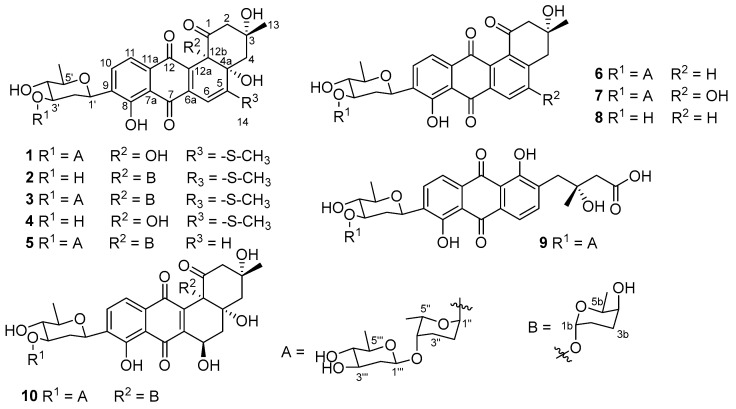
Structures of **1**–**10** isolated from *Streptomyces ardesiacus* 156VN-095.

**Figure 2:**
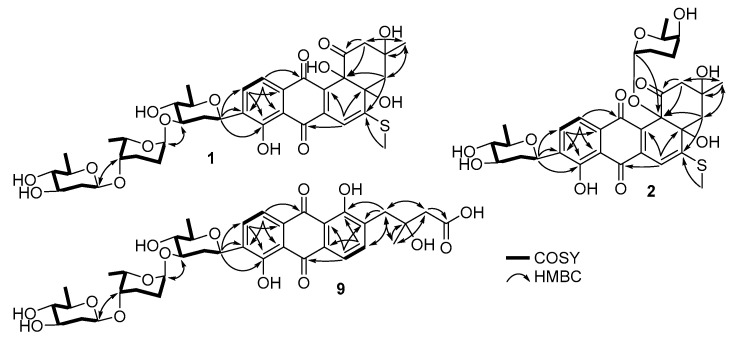
Key COSY and HMBC correlations for **1**, **2**, and **9**.

**Figure 3:**
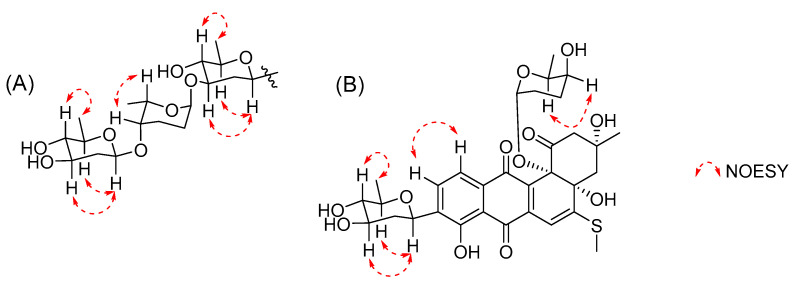
(**A**). Key NOESY correlations for trisaccharide moiety of **1** and **9**. (**B**). Key NOESY correlations for **2**.

**Figure 4:**
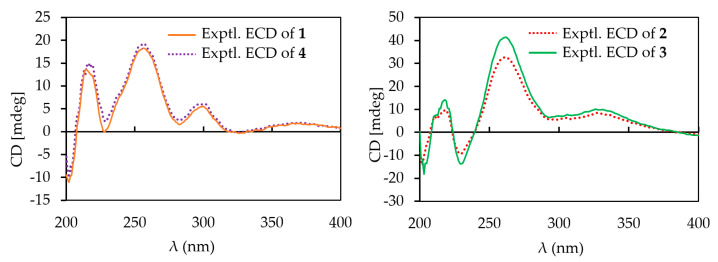
Experimental ECD spectra of **1**–**4**.

Compound **1** was isolated as a dark red powder, and the molecular formula of **1** was determined as C_38_H_48_O_15_S based on its HRESIMS peak at *m*/*z* 799.2612, [M + Na]^+^ (calcd. for C_38_H_48_O_15_SNa, 799.2612, Figure S8). The ^1^H NMR spectrum of **1** showed signals of three aromatic protons at *δ*_H_ 7.73 (d, *J* = 7.6 Hz, H-10), 7.42 (d, *J* = 6.8 Hz, H-11), and 6.42 (s, H-6); two anomeric protons at *δ*_H_ 4.96 (s, H-1″) and 4.57 (d, *J* = 9.6 Hz, H-1‴); nine oxygenated methines at *δ*_H_ 2.91–4.75; twelve methylene protons at *δ*_H_ 1.22–2.85; and signals of five methyl groups at *δ*_H_ 1.17–2.49 (Figure S2). The ^13^ C NMR data, in combination with HSQC spectrum, demonstrated signals of 13 sp^2^ carbons including three ketones at *δ*_C_ 206.6 (C-1), 190.1 (C-7), and 183.1 (C-12); three protonated aromatic carbons at *δ*_C_ 134.4 (C-10), 119.7 (C-11), and 106.5 (C-6); and seven non-protonated sp^2^ carbons at *δ*_C_ 115.3–165.8 (Figures S3 and S4). In addition, the ^13^C NMR spectrum also revealed signals of two anomeric carbons at *δ*_C_ 102.8 (C-1‴) and 95.2 (C-1″); nine protonated oxygenated methines at *δ*_C_ 67.8–78.4; three tertiary alcohols at *δ*_C_ 84.2 (C-4a), 79.5 (C-12b), and 77.3 (C-3); six methylenes at *δ*_C_ 25.4–53.3; and five methyl groups at *δ*_C_ 14.6–30.3. The ^1^H NMR spectrum of **1** was quite similar to that of urdamycin E (**3**), except for the obvious missing signals for a sugar moiety attached to C-12b, indicating **1** was a new derivative of **3** with one less sugar unit [12] as shown in Figure 1. Further detailed analysis of HMBC and COSY data (Figure 2 and Figures S5 and S6) confirmed the planar structure of **1**. The relative configuration of trisaccharide moiety was deduced by analysis of ^3^ *J*_H,H_ coupling constants and NOESY data. The strong NOESY correlations from H-1′ to H-3′ and H-5′ indicated H-1′, H-3′, and H-5′ had a co-facial relationship, and H-4′ was observed at *δ*_H_ 3.12 (t), with a large coupling constant (*J* = 8.9 Hz), indicating H-4′ had a diaxial relationship with H-3′ and H-5′ (Figure 3 and Figure S7). Thus, the first sugar was determined as *D*-olivose and, similarly, the third sugar was also determined as *D*-olivose. The strong NOESY correlation between H-4′′ and H_3_-5′′ and the lack of NOESY correlation from H-1′′ to H-5′′ identified the second sugar as *L*-rhodinose. Urdamycins are a group of angucycline glycosides firstly isolated from *Streptomyces fradiae* and the absolute stereochemistry of urdamycin A (**5**) was unambiguously determined by an X-ray analysis [13]. Urdamycin E (**3**) with an additional thiomethyl group (CH_3_S-) at C-5 position of urdamycin A (**5**) was also isolated from the same strain, and **3** was transformed into **5** by treatment with Raney nickel reagent [14]. Hydrolysis of **3** yielded urdamycinone E (**4**) [14]. These studies revealed that urdamycin A (**5**) and its congeners (**3** and **4**) have the same absolute stereochemistry. Therefore, the absolute stereochemistry of **1** was determined by comparison experimental ECD between **1** and **4** (Figure 4 and Figure S28), and by considering the similarity of ^1^H and ^13^C NMR data and biosynthetic correlation of **1** and **4**. Thus, the structure of **1** was determined as 12b-desrhodinosyl urdamycin E and named urdamycin W.

Compound **2** was also isolated as a dark red powder, the molecular formula of **2** was determined as C_32_H_38_O_12_S based on its HRESIMS peak at *m*/*z* 669.1984, [M + Na]^+^ (calcd. for C_32_H_38_O_12_SNa, 669.1982, Figure S15). The ^1^H NMR spectrum of **2** revealed signals of three aromatic protons at *δ*_H_ 7.84 (d, *J* = 7.8 Hz, H-10), 7.57 (d, *J* = 7.8 Hz, H-11), and 6.49 (s, H-6); an anomeric proton at *δ*_H_ 5.28 (s, H-1b); six oxygenated methines at *δ*_H_ 3.03–4.89; ten methylene protons at *δ*_H_ 1.38–2.78; and signals of four methyl groups at *δ*_H_ 0.55–2.48 (Figure S9). The ^13^C NMR data, in collaboration with HSQC spectrum, showed signals of 13 sp^2^ carbons including three ketocarbonyls at *δ*_C_ 204.7 (C-1), 190.3 (C-7), and 183.9 (C-12); three protonated aromatic carbons *δ*_C_ 134.4 (C-10), 120.1 (C-11), and 106.0 (C-6); and seven non-protonated sp^2^ carbons at *δ*_C_ 115.5–165.8 (Figures S10 and S11). Additionally, the ^13^C NMR spectrum also showed signals of an anomeric carbon at *δ*_C_ 95.7 (C-1b), six protonated oxygenated methines at *δ*_C_ 67.8–78.8, three tertiary alcohols at *δ*_C_ 84.7 (C-4a), 83.5 (C-12b), and 76.8 (C-3), five methylenes at *δ*_C_ 24.2–55.0, and four methyl groups at *δ*_C_ 14.6–30.0 (Figure S10). The ^1^H NMR spectrum of **2** was similar to that of urdamycin V, except for the obvious missing signals of a doublet anomeric proton at *δ*_H_ 4.98 and a doublet methyl group at *δ*_H_ 1.14 in urdamycin V, indicating **2** was a new derivative of urdamycin V with a missing sugar moiety attached to C-3′ position [12]. Further detailed analysis of HMBC and COSY data confirmed the planar structure of **2** as depicted in Figure 1 (Figures S12 and S13). Two sugar units were determined as *D*-olivose and *L*-rhodinose by a similar procedure for **1** (Figure S14). The absolute stereochemistry of **2** was determined by comparison of its experimental ECD spectrum with that of **3** (Figure 4 and Figure S29). Thus, the structure of **2** was determined as 3′-desrhodinosyl urdamycin V and named urdamycin X.

Compound **9** was isolated as an orange powder. The molecular formula of **9** was determined as C_37_H_46_O_15_ based on its HRESIMS peak at *m*/*z* 753.2734, [M + Na]^+^ (calcd. for C_37_H_46_O_15_Na, 753.2734, Figure S27), with two hydroxy groups (-OH) more than that of **6** (urdamycin B). The ^1^H NMR spectrum of **9** showed a similar pattern to that of **6**, and the obvious differences were the upfield-shifted chemical shift values of H-5 and H_3_-13 (Figures S16 and S22)). The ^13^C NMR spectrum of **9** showed an additional carbonyl signal at *δ*_C_ 175.7 and a missed ketone signal at *δ*_C_ 196.7 [14], indicating **9** was a new derivative of **6** with a ring-opening type of ring A and the ketone was hydrolyzed to a carboxylic acid (grincamycin type, Figures S17 and S23) [15]. The planar structure of **9** was further confirmed by detailed analysis of HSQC, ^1^H-^1^H COSY, and HMBC data (Figures S18–S20 and S24–S26). The relative configuration of the trisaccharide moiety was determined to be the same as that of **1** by analysis of NOESY data and ^3^*J*_H,H_ coupling constants (Figures S21). The absolute configuration of **9** was the same as that of other gricamycin derivatives by considering the biosynthetic correlation and comparison of experimental ECD spectra of **9** with grincamycin L (Figure S30) [15]. Thus, the structure of **9** was determined as depicted in Figure 1 and named grincamycin U [16].

The structures of known compounds were identified as urdamycin E (**3**) [12], urdamycinone E (**4**) [14], urdamycin A (**5**) [13], urdamycin B (**6**) [14], 5-hydroxyurdamycin B (**7**) [17], urdamycinone B (**8**) [14], and urdamycin F (**10**) [14] by comparison of their spectroscopic data with those reported in the literature (Figures S31–S37).

Since previously described angucyline glycosides showed anti-Gram-positive bacterial or cytotoxic activities [9], the new compounds (**1**, **2**, and **9**) were primarily evaluated for their anti-bacterial activity against three Gram-positive bacterial strains (Table 1). The tested compounds showed selective anti-microbial activity, and of them, **1** showed the strongest activity against *Bacillus substilis* (KCTC 1021) with a MIC value of 8.0 µg/mL.

Compounds **1–3** and **9** were also tested for their cytotoxicity against six cancer cell lines (PC-3 (prostate), NCI-H23 (lung), HCT-15 (colon), NUGC-3 (stomach), ACHN (renal), and MDA-MB-231 (breast)). All compounds showed cytotoxic effect with a different tendency (Table 2 and Figure S38). Compound **1** showed the strongest cytoxicity against all tested cell lines, which was more potent than the positive control (adriamycin). The cytotoxic and anti-bacterial results indicated that a longer saccharide chain at C-3′ could enhance the activities (**1** and **2**) and ring-opening type (**9**) led to a significant reduction in their biological effects.

## 3. Materials and Methods

### 3.1. General Experimental Procedures

The 1D and 2D NMR spectra were recorded using a Bruker AVANCE III 600 spectrometer with a 3 mm probe operating at 600 MHz (^1^H) and 150 MHz (^13^C). HRESIMS data were acquired by a Waters SYNPT G2 Q-TOF mass spectrometer at the Korea Basic Science Institute (KBSI) in Cheongju, Korea. UV spectra were measured by a Shimadzu UV-1650PC spectrophotometer. ECD spectra were obtained on a JASCO J-1500 polarimeter at the Center for Research Facilities, Changwon National University, Changwon, Korea. IR spectra were recorded on a JASCO FT/IR-4100 spectrophotometer. HPLC was carried out using a PrimeLine binary pump coupled with a Shodex RI-101 refractive index detector and S3210 variable UV detector. Columns used for HPLC were YMC-Triart C_18_ (250 mm × 10 mm, 5 μm and 250 mm × 4.6 mm, 5 μm). Reversed-phase silica gel (YMC-Gel ODS-A, 12 nm, S-75 μm) was used for open-column chromatography. Mass culture was conducted using a Fermentec 100 L fermenter. All solvents were either HPLC grade or distilled prior to use.

### 3.2. Bacterial Strain, Fermentation, and Isolation of 1–10 from Streptomyces ardesiacus 156VN-095

The strain 156VN-095 was isolated from an unidentified sponge collected near Nha Trang Bay, Vietnam, in June 2015. The strain was identified as *Streptomyces ardesiacus* based on 16S rRNA gene sequence analysis (GenBank accession number OP604346) by Macrogen Inc. (Seoul, Korea). The seed and mass cultures of the strain were conducted in Bennett’s modified medium (BN broth, 0.5% glucose, 0.05% yeast extract, 0.1% tryptone, 0.05% beef extract, 0.25% glycerol, and 3.2% sea salt). The strain was grown on BN agar plates at 28 °C for 7 days. The bacterial spores were then inoculated into BN broth medium (50 mL) in a 100 mL flask and incubated in a rotation shaker (140 rpm) at 28 °C for 4 days. An aliquot (10 mL) of the seed culture was then transferred into the BN broth medium (1.0 L) in a 2.0 L flask and grown under the same afore-mentioned conditions. The culture was then inoculated into a 100 L fermenter filled with 70 L of BN medium and cultured for 11 days and then harvested. The culture broth and the cells were separated by centrifugation and the broth was extracted with an equal volume of EtOAc, twice. The organic layer was evaporated under reduced pressure to yield a crude extract (6.0 g). The extract was fractionated into 10 fractions (F1 to F10) by liquid vacuum chromatography on an OSD column using a stepwise elution of 10 to 100% MeOH in H_2_O. The F6 fraction was subjected to a semi-preparative HPLC (YMC-PackODS-A, 250 × 10 mm i.d., 5 μm, flow rate 2.0 mL/min) with an isocratic elution of 53% MeOH in H_2_O to obtain compounds **1** (3.0 mg, *t_R_* = 48.5 min), **3** (4.0 mg, *t_R_* = 54.0 min), **5** (4.2 mg, *t_R_* = 33.2 min), **6** (5.0 mg, *t_R_* = 75.0 min), and **7** (1.0 mg, *t_R_* = 80.3 min) and subfraction F6-1. The subfraction F6-1 was repurified by a semi-preparative HPLC (YMC-PackODS-A, 250 × 10 mm i.d., 5 μm, flow rate 2.0 mL/min) with an isocratic elution of 23% MeCN in H_2_O to obtain compounds **10** (1.3 mg, *t_R_* = 26.5 min), **8** (7.0 mg, *t_R_* = 37.0 min), **4** (1.0 mg, *t_R_* = 40.2 min), and **2** (3.0 mg, *t_R_* = 50.6 min). Compound **9** (3.2 mg, *t_R_* = 30.1 min) was isolated from the F8 fraction by a semi-preparative HPLC (YMC-PackODS-A, 250 × 10 mm i.d., 5 μm, flow rate 2.0 mL/min) with an isocratic elution of 75% MeOH in H_2_O.

Urdamycin W (**1**): dark red powder; IR ν_max_ 3398, 2929, 1632, 1515, 1430, 1367, 1293, 1063 cm^−1^; UV (MeOH) λ_max_ (log ε) 291 (4.7), 475 (4.2) nm; HRESIMS *m*/*z* 799.2612, [M + Na]^+^ (calcd. for C_38_H_48_O_15_SNa, 799.2612), ^1^ H NMR (CD_3_OD, 600 MHz) and ^13^ C NMR (CD_3_OD, 150 MHz) see Table 3.

Urdamycin X (**2**): dark red powder; IR ν_max_ 3417, 2929, 1632, 1515, 1430, 1299, 1088 cm^−1^; UV (MeOH) λ_max_ (log ε) 298 (4.9), 470 (4.3) nm; HRESIMS *m*/*z* 669.1984, [M + Na]^+^ (calcd. for C_32_H_38_O_12_SNa, 669.1982), ^1^H NMR (CD_3_OD, 600 MHz) and ^13^C NMR (CD_3_OD, 150 MHz) see Table 3.

Grincamycin U (**9**): orange powder; IR ν_max_ 3396, 2929, 1628, 1430, 1371, 1257, 1070 cm^−1^; UV (MeOH) λ_max_ (log ε) 230 (4.8), 255 (3.9), 442 (3.2) nm; HRESIMS *m*/*z* 753.2734, [M + Na]^+^ (calcd. for C_37_H_46_O_15_Na, 753.2734), ^1^H NMR (pyridine-*d*_5_, 600 MHz) and ^13^C NMR (pyridine-*d*_5_, 150 MHz) see Table 3.

### 3.3. Antibacterial Assay

The antimicrobial assay of **1**, **2**, and **9** was conducted using a standard broth dilution assay. Difco^TM^ Mueller–Hinton broth (BD, 275730) was used for determination of MIC values. Compounds **1**, **2**, and **9** were tested against three Gram-positive bacteria including, *Staphylococcus aureus* (KCTC 1927), *Micrococcus luteus* (KCTC 1915), and *Bacillus subtilis* (KCTC 1021). The tested compounds were prepared in the range of 0.5–256 µg/mL in 96-microtiter plates by a serial double dilution. An overnight culture broth of each strain was dispensed in sterilized 0.9% saline to an inoculum density of 5 × 10^5^ cfu by comparison with a McFarland standard [18]. The diluted culture broth (100 µL) was added to each dilution of the tested compounds (**1**, **2**, and **9**, 100 µL), in the plate to yield final concentrations from 0.25 to 128 µg/mL. The plates were maintained for 24 h at 37 °C. The MIC value is the lowest concentration at which the microorganism did not demonstrate visible growth, as indicated by the presence of turbidity. Kanamycin was used as a positive control. All experiments were conducted twice to check reproducibility.

### 3.4. Cytotoxicity Test by SRB Assay

The SRB cytotoxicity test for **1**–**3**, and **9** was performed as previously described [19]. Cancer cell lines were obtained from Japanese Cancer Research Resources Bank (JCRB) (NUGC-3, JCRB Cell Bank/Cat. #JCRB0822) and American Type Culture Collection (ATCC) (PC-3, ATCC/Cat. #CRL-1435; MDA-MB-231, ATCC/Cat. #HTB-26; ACHN, ATCC/Cat. #CRL-1611; NCI-H23, ATCC/Cat. #CRL-5800; HCT-15, ATCC/Cat. #CCL-225).

### 3.5. Statistical Analysis

Statistical analysis was evaluated by one-way ANOVA followed by Dunnett’s t-test and the GI_50_ values were determined by the software of GraphPad Prism 8 (GraphPad Software Inc., San Diego, CA, USA).

## 4. Conclusions

In conclusion, we have isolated 10 angucycline glycosides from the culture broth of *Streptomyces ardesiacus* 156VN-095 and three of them were new compounds (**1**, **2**, and **9**). The structures of the new metabolites were elucidated by spectroscopic analysis and comparison of their experimental data with those reported in the literature. The new compounds showed selective anti-bacterial effects against three Gram-positive bacterial strains and significant cytotoxicity against a panel of cancer cell lines with a different potency. Among them, **1** showed the strongest activities against all the tested cell lines with GI_50_ values of 0.019–0.104 µM. These results expanded biochemical diversities of naturally occurring angucycline glycosides.

## Figures and Tables

**Table 1:** Antibacterial activity of **1**, **2**, and **9**.

MIC (µg/mL)			
	*B. subtilis* KCTC 1021	*Micrococcus luteus* KCTC 1915	*Staphylococcus aureus* KCTC 1927
**1**	8.0	64.0	32.0
**2**	>128	64.0	64.0
**9**	32.0	>128	>128
Kanamycin	0.25	1.0	0.5

**Table 2:** Growth inhibition (GI_50_, *µ*M) of **1**–**3**, and **9** against human cancer cell lines.

Compounds	1	2	3	9	Adr.
ACHN	0.104 ± 0.012	0.093 ± 0.004	0.060 ± 0.001	3.422 ± 0.357	0.140 ± 0.009
HCT-15	0.075 ± 0.012	0.150 ± 0.015	0.095 ± 0.037	3.886 ± 0.351	0.162 ± 0.012
MDA-MB-231	0.033 ± 0.008	0.077 ± 0.017	0.093 ± 0.005	3.500 ± 0.472	0.162 ± 0.000
NCI-H23	0.031 ± 0.002	0.050 ± 0.004	0.036 ± 0.002	3.245 ± 0.179	0.145 ± 0.003
NUGC-3	0.019 ± 0.003	0.028 ± 0.006	0.030 ± 0.006	3.037 ± 0.045	0.151 ± 0.014
PC-3	0.022 ± 0.006	0.103 ± 0.002	0.062 ± 0.012	2.750 ± 0.344	0.148 ± 0.005

Adr., Adriamycin as a positive control. GI_50_ values are the concentration corresponding to 50% growth inhibition.

**Table 3:** ^1^H and ^13^C NMR spectroscopic data of **1**, **2**, and **9** (600 MHz for ^1^H and 150 MHz for ^13^C).

1 ^a^			2 ^a^			9 ^b^		
Pos.	*δ*_H,_ Mult (*J* in Hz)	*δ*_C_	Pos.	*δ*_H,_ Mult (*J* in Hz)	*δ*_C_	Pos.	*δ*_H,_ Mult (*J* in Hz)	*δ*_C_
1		206.6	1		204.7	1		175.7
2	2.85, d (13.0) 2.66, d (13.3)	53.3	2	2.78, d (13.1) 2.56, dd (13.1, 2.6)	55.0	2	3.00, m	47.0
3		77.3	3		76.8	3		72.4
4	2.13, s	46.6	4	2.21, d (14.9) 2.06, dd (15.0, 2.6)	46.1	4	3.44, m	41.4
4a		84.2	4a		84.7	4a		136.6
5		165.8	5		165.8	5	8.03, d (7.6)	140.6
6	6.42, s	106.5	6	6.49, s	106.0	6	7.88, d (7.6)	119.3
6a		139.6	6a		138.7	6a		132.3
7		190.1	7		190.3	7		188.9
7a		115.3	7a		115.5	7a		116.3
8		158.7	8		158.7	8		159.5
9		138.5	9		138.6	9		139.4
10	7.73, d (7.6)	134.4	10	7.84, d (7.8)	134.4	10	8.09, d (7.8)	134.1
11	7.42, d (6.8)	119.7	11	7.57, d (7.8)	120.1	11	7.98, d (7.8)	119.7
11a		132.3	11a		132.6	11a		132.7
12		183.1	12		183.9	12		188.8
12a		134.7	12a		135.4	12a		116.3
12b		79.5	12b		83.5	12b		162.3
13	1.24, s	30.3	13	1.21, s	30.0	13	1.69, s	28.1
14	2.49, s	14.6	14	2.48, s	14.6	14		
1′	4.75, d (11.1)	72.3	1′	4.89, d (11.0)	72.4	1′	5.11, d (11.2)	72.1
2′	2.45, m 1.22, m	37.6	2′	2.40, dd (12.7, 4.7) 1.38, m	41.1	2′	2.81, dd (12.0, 3.1) 1.61, m	37.7
3′	3.73, m	77.8	3′	3.69, m	73.6	3′	4.21, m	78.2
4′	3.12, t (8.9)	76.8	4′	3.03, t (8.9)	78.8	4′	3.64, m	76.4
5′	3.45, m	77.7	5′	3.44, dq (12.2, 6.1)	77.8	5′	3.81, m	77.8
6′	1.38, d (5.9)	18.9	6′	1.37, d (6.2)	18.6	6′	1.69, d (6.3)	19.4
1″	4.96, s	95.2	1b	5.28, s	95.7	1″	5.27, s	95.1
2″	2.05, m 1.44, m	25.6	2b	1.86, m	24.2	2″	2.38, m 2.25, m	25.7
3″	2.05, m 1.94, m	25.4	3b	2.01, m 1.58, dd (2.5, 13.2)	26.5	3″	2.25, m 1.62, m	25.7
4″	3.55, s	77.7	4b	3.35, s	67.8	4″	3.67, s	76.9
5″	4.24, q (6.4)	67.8	5b	3.64, q (6.5)	68.3	5″	4.67, q (6.0)	67.3
6″	1.17, d (6.4)	17.4	6b	0.55, d (6.6)	17.0	6″	1.38, d (6.4)	17.9
1‴	4.57, d (9.6)	102.8				1‴	4.80, d (9.6)	102.9
2‴	2.20, dd (12.3, 4.9) 1.55, m	40.6				2‴	2.66, dd (12.1, 4.3) 2.17, m	41.4
3‴	3.50, m	72.3				3‴	4.08, m	72.5
4‴	2.91, t (9.0)	78.4				4‴	3.56, t (8.7)	78.9
5‴	3.23, dq (12.5, 6.1)	73.2				5‴	3.64, m	73.4
6‴	1.26, d (6.0)	18.4				6‴	1.61, d (6.1)	19.2

^a^ measured in methanol-*d*_4;_^b^ measured in pyridine-*d*_5._

## Data Availability

The data presented in the article are available in the Supplementary Materials.

## Supplementary Materials

The following supporting information can be downloaded at: https://www.mdpi.com/article/10.3390/ijms232213779/s1.

## References

[1] Quinn G.A., Banat A.M., Abdelhameed A.M., Banat I.M. (2020). *Streptomyces* from traditional medicine: Sources of new innovations in antibiotic discovery. J. Med. Microbiol..

[2] Mast Y., Stegmann E. (2019). Actinomycetes: The Antibiotics Producers. Antibiotics.

[3] Saito S., Xiaohanyao Y., Zhou T., Nakajima-Shimada J., Tashiro E., Triningsih D.W., Harunari E., Oku N., Igarashi Y. (2022). Phytohabitols A–C, *δ*-Lactone-Terminated Polyketides from an Actinomycete of the Genus *Phytohabitans*. J. Nat. Prod..

[4] Hubert J., Nuzillard J.-M., Renault J.-H. (2017). Dereplication strategies in natural product research: How many tools and methodologies behind the same concept?. Phytochem. Rev..

[5] Voser T.M., Campbell M.D., Carroll A.R. (2021). How different are marine microbial natural products compared to their terrestrial counterparts?. Nat. Prod. Rep..

[6] Risdian C., Mozef T., Wink J. (2019). Biosynthesis of Polyketides in *Streptomyces*. Microorganisms.

[7] Lacey H.J., Rutledge P.J. (2022). Recently Discovered Secondary Metabolites from *Streptomyces* Species. Molecules.

[8] Kharel M.K., Pahari P., Shepherd M.D., Tibrewal N., Nybo S.E., Shaaban K.A., Rohr J. (2012). Angucyclines: Biosynthesis, mode-of-action, new natural products, and synthesis. Nat. Prod. Rep..

[9] Hulst M.B., Grocholski T., Neefjes J.J.C., van Wezel G.P., Metsä-Ketelä M. (2022). Anthracyclines: Biosynthesis, engineering and clinical applications. Nat. Prod. Rep..

[10] Hu Y., Nie Q.-Y., Pan H.-X., Tang G.-L., Liu H.-W., Begley T.P. (2020). 1.07-Bacterial Type II Polyketide Synthases. Comprehensive Natural Products III.

[11] Rohr J., Thiericke R. (1992). Angucycline group antibiotics. Nat. Prod. Rep..

[12] Dan V.M., Vinodh J.S., Sandesh C.J., Sanawar R., Lekshmi A., Kumar R.A., Santhosh Kumar T.R., Marelli U.K., Dastager S.G., Pillai M.R. (2020). Molecular networking and whole-genome analysis aid discovery of an angucycline that inactivates mTORC1/C2 and induces programmed cell death. ACS Chem. Biol..

[13] Drautz H., Zähner H., Rohr J., Zeeck A. (1986). Metabolic products of microorganisms. 234. Urdamycins, new angucycline antibiotics from *Streptomyces fradiae*. I. Isolation, characterization and biological properties. J. Antibiot..

[14] Rohr J., Zeeck A. (1987). Metabolic products of microorganisms. 240 Urdamycins, new angucycline antibiotics from *Streptomyces fradiae*. II Structural studies of urdamycins B to F. J. Antibiot..

[15] Yang L., Hou L.K., Li H.Y., Li W.L. (2020). Antibiotic angucycline derivatives from the deepsea-derived *Streptomyces lusitanus*. Nat. Prod. Res..

[16] Shang Z., Ferris Z.E., Sweeney D., Chase A.B., Yuan C., Hui Y., Hou L., Older E.A., Xue D., Tang X. (2021). Grincamycins P–T: Rearranged Angucyclines from the Marine Sediment-Derived *Streptomyces* sp. CNZ-748 Inhibit Cell Lines of the Rare Cancer Pseudomyxoma Peritonei. J. Nat. Prod..

[17] Rohr J., Schoenewolf M., Udvarnoki G., Eckardt K., Schumann G., Wagner C., Beale J.M., Sorey S.D. (1993). Investigations on the biosynthesis of the angucycline group antibiotics aquayamycin and the urdamycins A and B. Results from the structural analysis of novel blocked mutant products. J. Org. Chem..

[18] Appendino G., Gibbons S., Giana A., Pagani A., Grassi G., Stavri M., Smith E., Rahman M.M. (2008). Antibacterial cannabinoids from *Cannabis sativa*: A structure-activity study. J. Nat. Prod..

[19] Choi B.-K., Trinh P.T.H., Lee H.-S., Choi B.-W., Kang J.S., Ngoc N.T.D., Van T.T.T., Shin H.J. (2019). New Ophiobolin Derivatives from the Marine Fungus *Aspergillus flocculosus* and Their Cytotoxicities against Cancer Cells. Mar. Drugs.

