# Supplementary material for: New Angucycline Glycosides from a Marine-Derived Bacterium Streptomyces ardesiacus
Source: Int J Mol Sci. 2022 Nov 9;23(22):13779. doi: 10.3390/ijms232213779 (PMC9698790; doi:10.3390/ijms232213779)
Supplement: Supplementary file 1 [file ijms-23-13779-s001.zip › ijms-2003943-supplementary.pdf]

New Angucycline Glycosides from a Marine-Derived Bacterium *Streptomyces ardesiacus*

Cao Van Anh [1,2], Joo-Hee Kwon[3], Jong Soon Kang [3], Hwa-Sun Lee [1], Chang-Su Heo [1,2] and Hee Jae Shin [1,2],*

[1] Marine Natural Products Chemistry Laboratory, Korea Institute of Ocean Science and Technology, 385 Haeyang-ro, Yeongdo-gu, Busan 49111, Korea; caovananh@kiost.ac.kr (C.V.A.); hwasunlee@kiost.ac.kr (H.-S.L.); science30@kiost.ac.kr (C.-S.H.)

[2] Department of Marine Biotechnology, University of Science and Technology (UST), 217 Gajungro, Yuseong-gu, Daejeon 34113, Korea

[3] Laboratory Animal Resource Center, Korea Research Institute of Bioscience and Biotechnology, 30 Yeongudanjiro, Cheongju 28116, Korea; juhee@kribb.re.kr (J.-H.K.); kanjon@kribb.re.kr (J.S.K.)

* Correspondence: shinhj@kiost.ac.kr; Tel.: +82-51-664-3341; Fax: +82-51-664-3340

Contents

Figure S1. Structures of **1-10** isolated from *Streptomyces ardesiacus* 156VN-095. 4
Figure S2. ^{1}H NMR spectrum of **1**. 5
Figure S3. ^{13}C NMR spectrum of **1**. 6
Figure S4. HSQC spectrum of **1**. 7
Figure S5. ^{1}H-^{1}H COSY spectrum of **1**. 8
Figure S6. HMBC spectrum of **1**. 9
Figure S7. NOESY spectrum of **1**. 10
Figure S8. HRESIMS data of **1**. 11
Figure S9. ^{1}H NMR spectrum of **2**. 12
Figure S10. ^{13}C NMR spectrum of **2**. 13
Figure S11. HSQC spectrum of **2**. 14
Figure S12. ^{1}H-^{1}H COSY spectrum of **2**. 15
Figure S13. HMBC spectrum of **2**. 16
Figure S14. NOESY spectrum of **2**. 17
Figure S15. HRESIMS data of **2**. 18
Figure S16. ^{1}H NMR spectrum of **9** (pyridine-d_5). 19
Figure S17. ^{13}C NMR spectrum of **9** (pyridine-d_5). 19
Figure S18. HSQC spectrum of **9** (pyridine-d_5). 20
Figure S19. ^{1}H-^{1}H COSY spectrum of **9** (pyridine-d_5). 21
Figure S20. HMBC spectrum of **9** (pyridine-d_5). 22
Figure S21. NOESY spectrum of **9** (pyridine-d_5). 23

Figure S22. ^{1}H NMR spectrum of **9** (DMSO-d_6)........ 24
Figure S23. ^{13}C NMR spectrum of **9** (DMSO-d_6)........ 25
Figure S24. HSQC spectrum of **9** (DMSO-d_6). 26
Figure S25. ^{1}H-^{1}H COSY spectrum of **9** (DMSO-d_6)........ 27
Figure S26. HMBC spectrum of **9** (DMSO-d_6). 28
Figure S27. HRESIMS data of **9**........ 29
Figure S28. Comparison of ECD spectra between **1** and **4**........ 30
Figure S29. Comparison of ECD spectra between **2** and **3**........ 31
Figure S30. Comparison of ECD spectra between grincamycin U (**9**) and grincamycin L........ 32
Figure S31. ^{1}H NMR spectrum of **3**........ 33
Figure S32. ^{1}H NMR spectrum of **4**........ 34
Figure S33. ^{1}H NMR spectrum of **5**........ 35
Figure S34. ^{1}H NMR spectrum of **6**........ 36
Figure S35. ^{1}H NMR spectrum of **7**........ 37
Figure S36. ^{1}H NMR spectrum of **8**........ 38
Figure S37. ^{1}H NMR spectrum of **10**........ 39
Figure S38. Results of the cytotoxicity test for **1-3**, and **9**........ 41

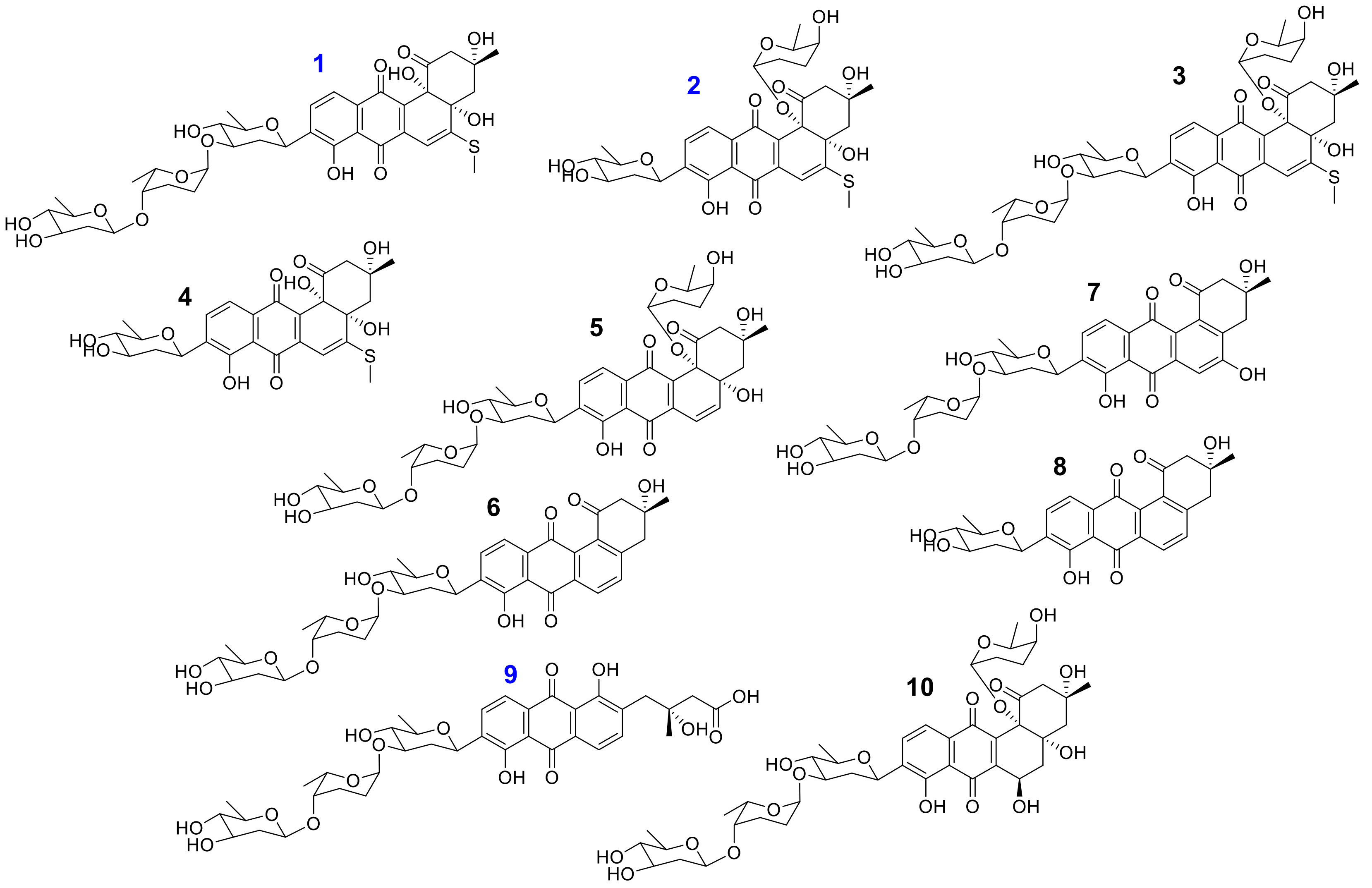

Figure S1. Structures of **1-10** isolated from *Streptomyces ardesiacus* 156VN-095.

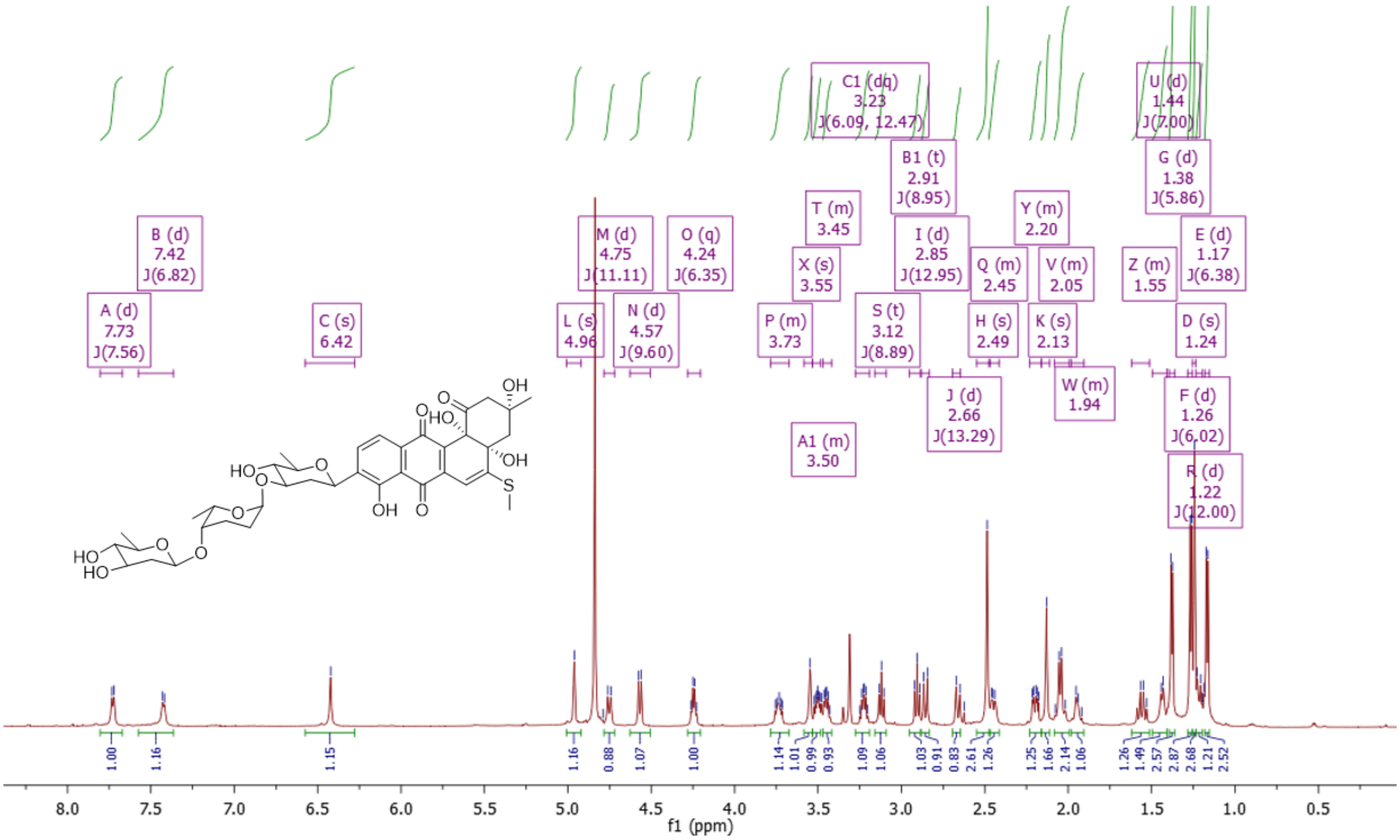

Figure S2. ^{1}H NMR spectrum of **1**.

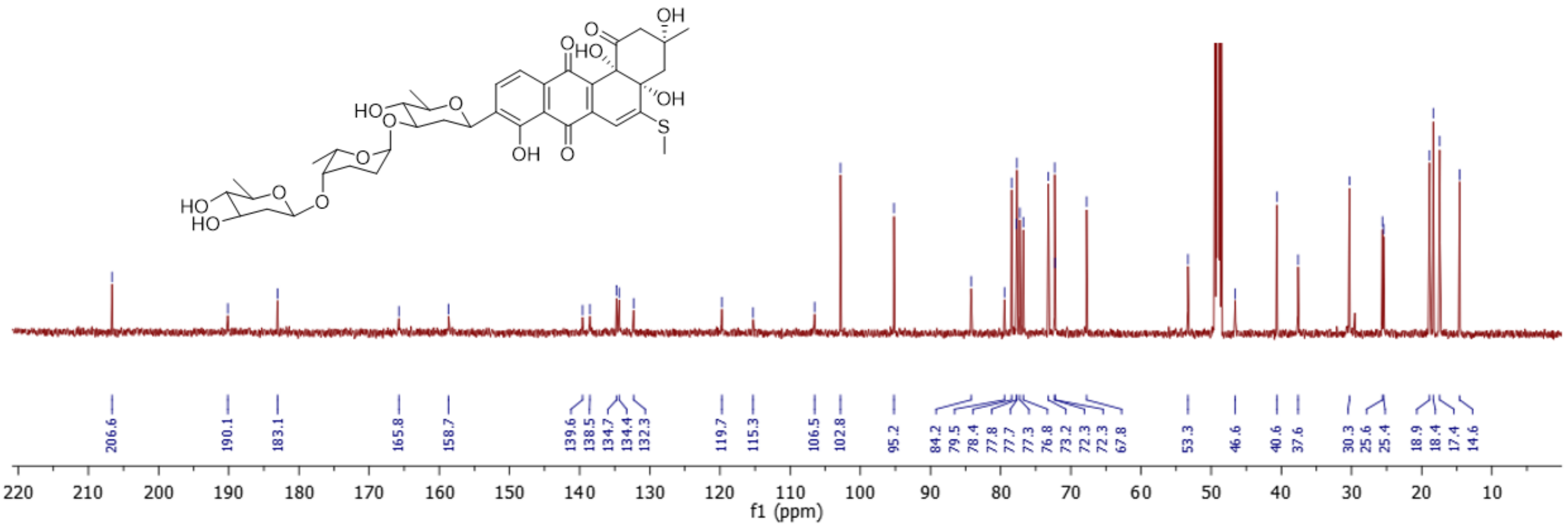

Figure S3. ^{13}C NMR spectrum of **1**.

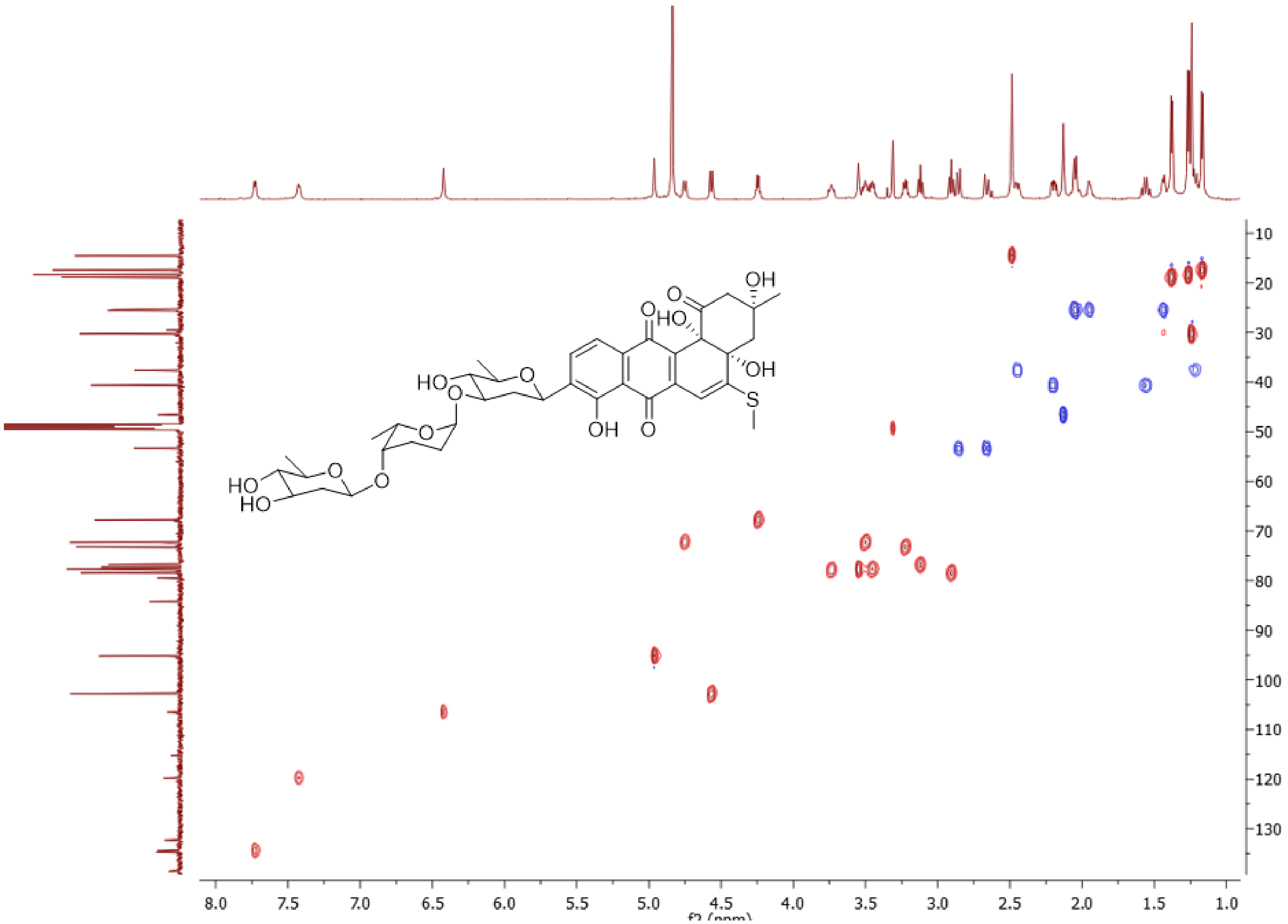

Figure S4. HSQC spectrum of **1**.

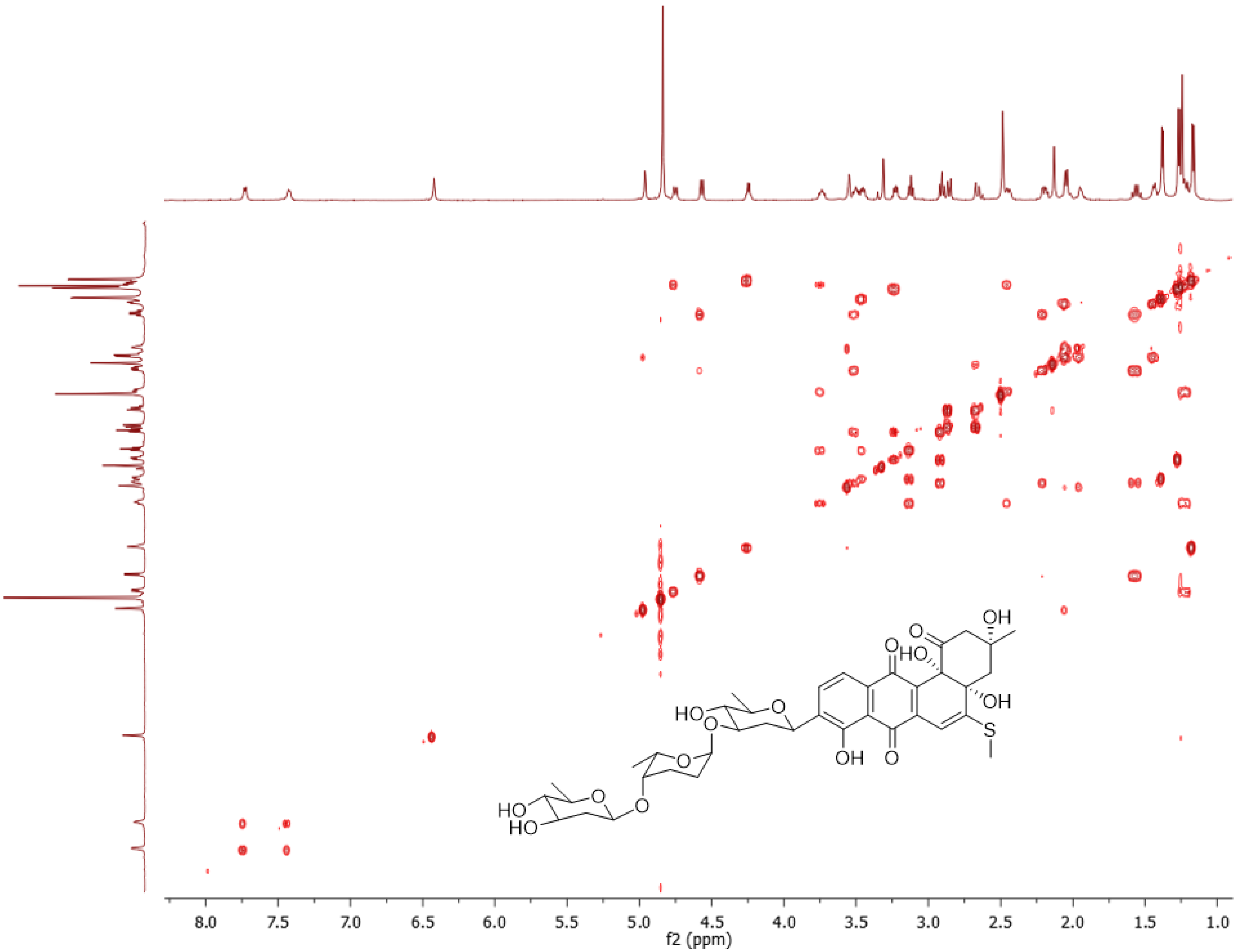

Figure S5. ^{1}H-^{1}H COSY spectrum of **1**.

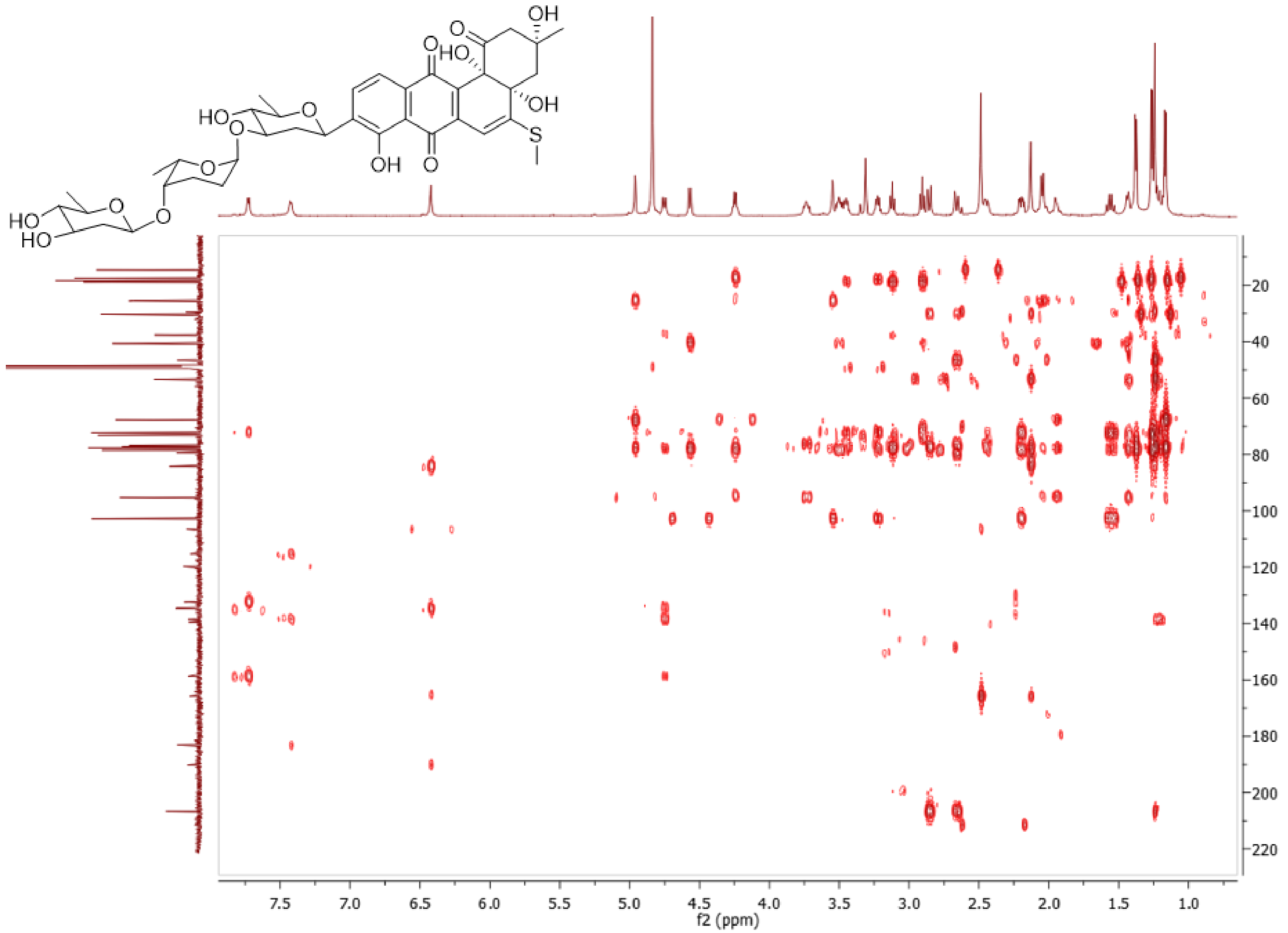

Figure S6. HMBC spectrum of **1**.

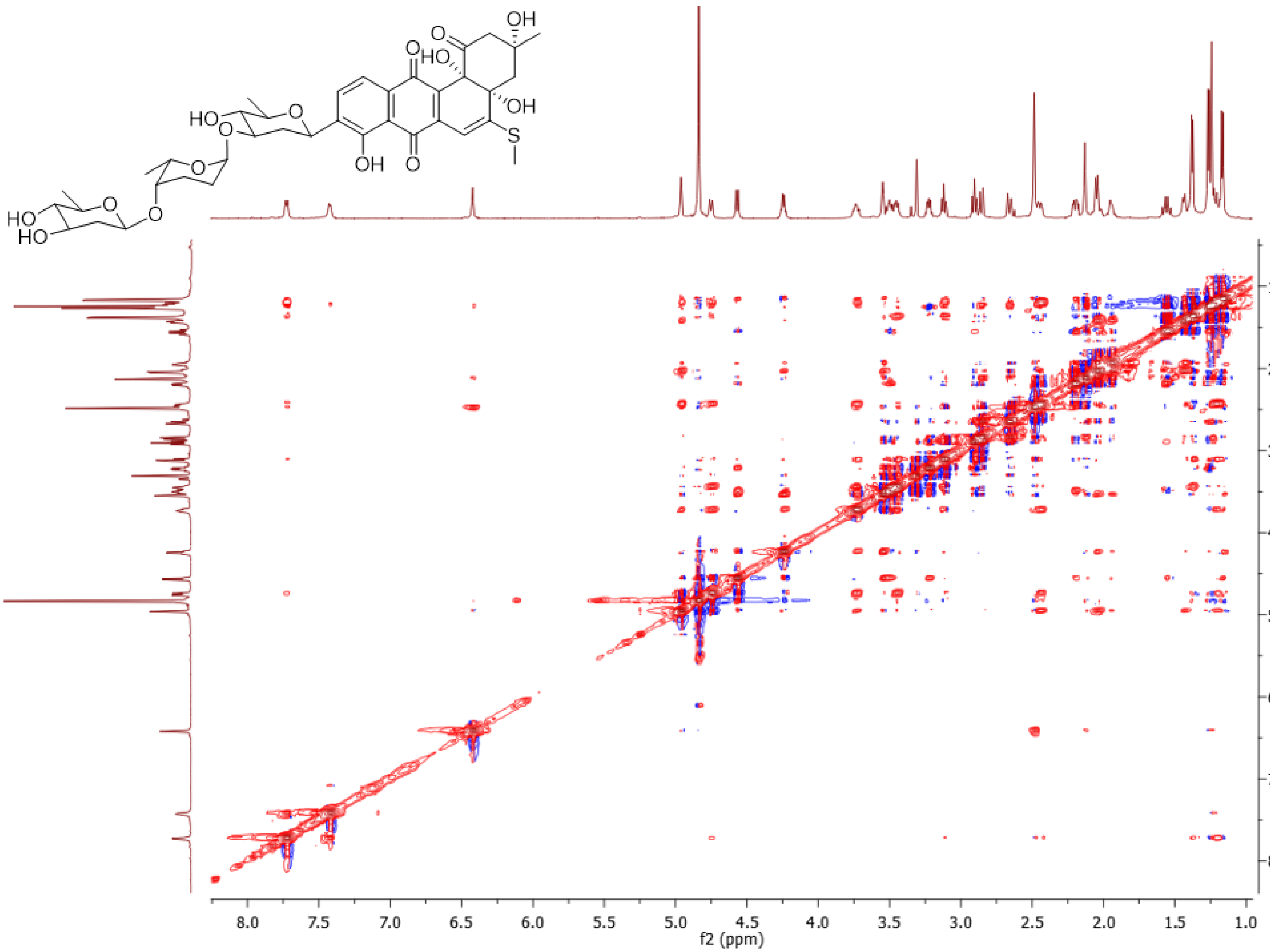

Figure S7. NOESY spectrum of **1**.

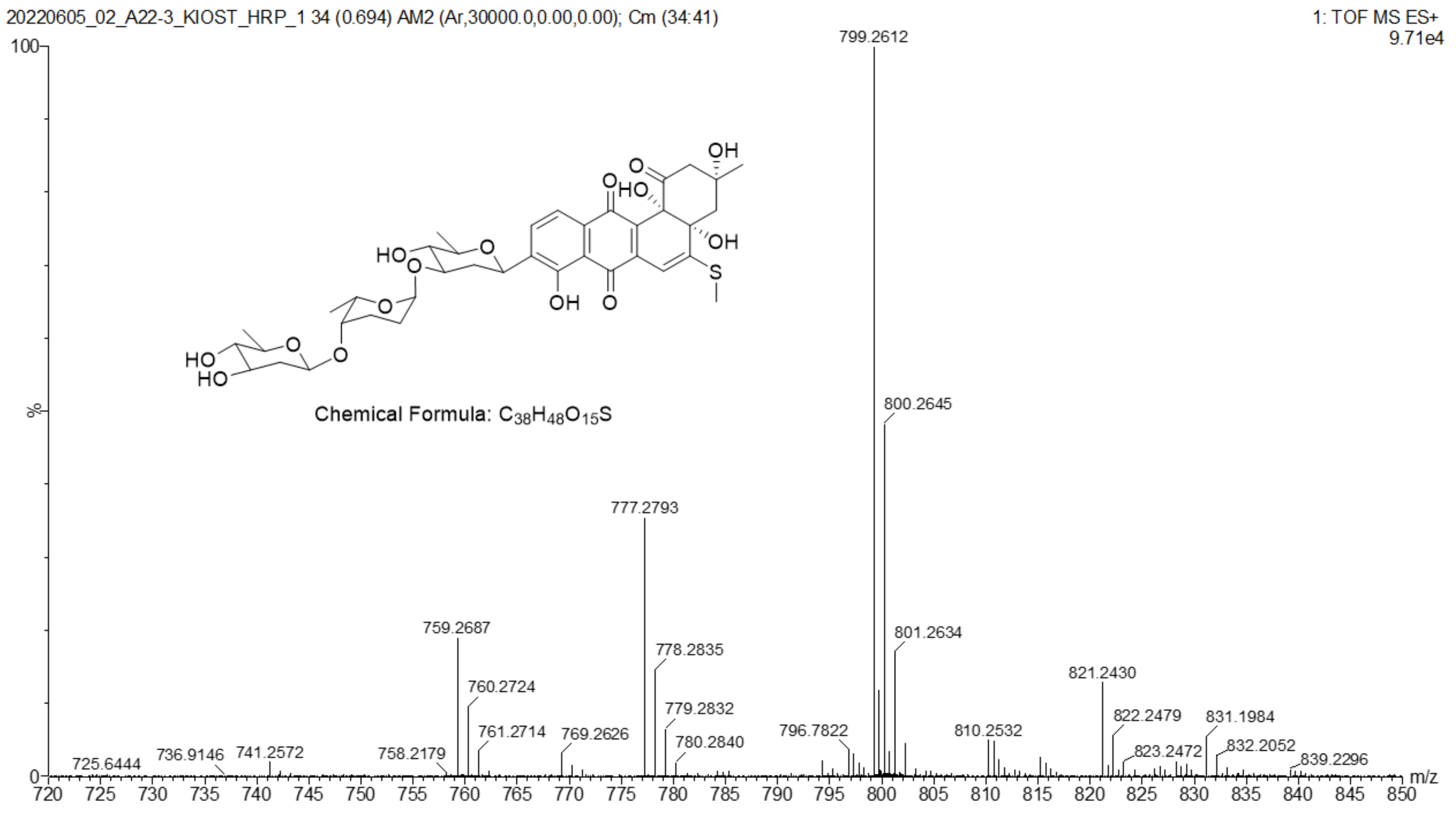

Mass	Calc. Mass	mDa	PPM	DBE	i-FIT	Norm	Conf(%)	Formula
799.2612	799.2612	0.0	0.0	14.5	468.9	0.668	51.27	C38 H48 O15 Na S
	799.2636	-2.4	-3.0	17.5	468.9	0.719	48.73	C40 H47 O15 S

Figure S8. HRESIMS data of **1**.

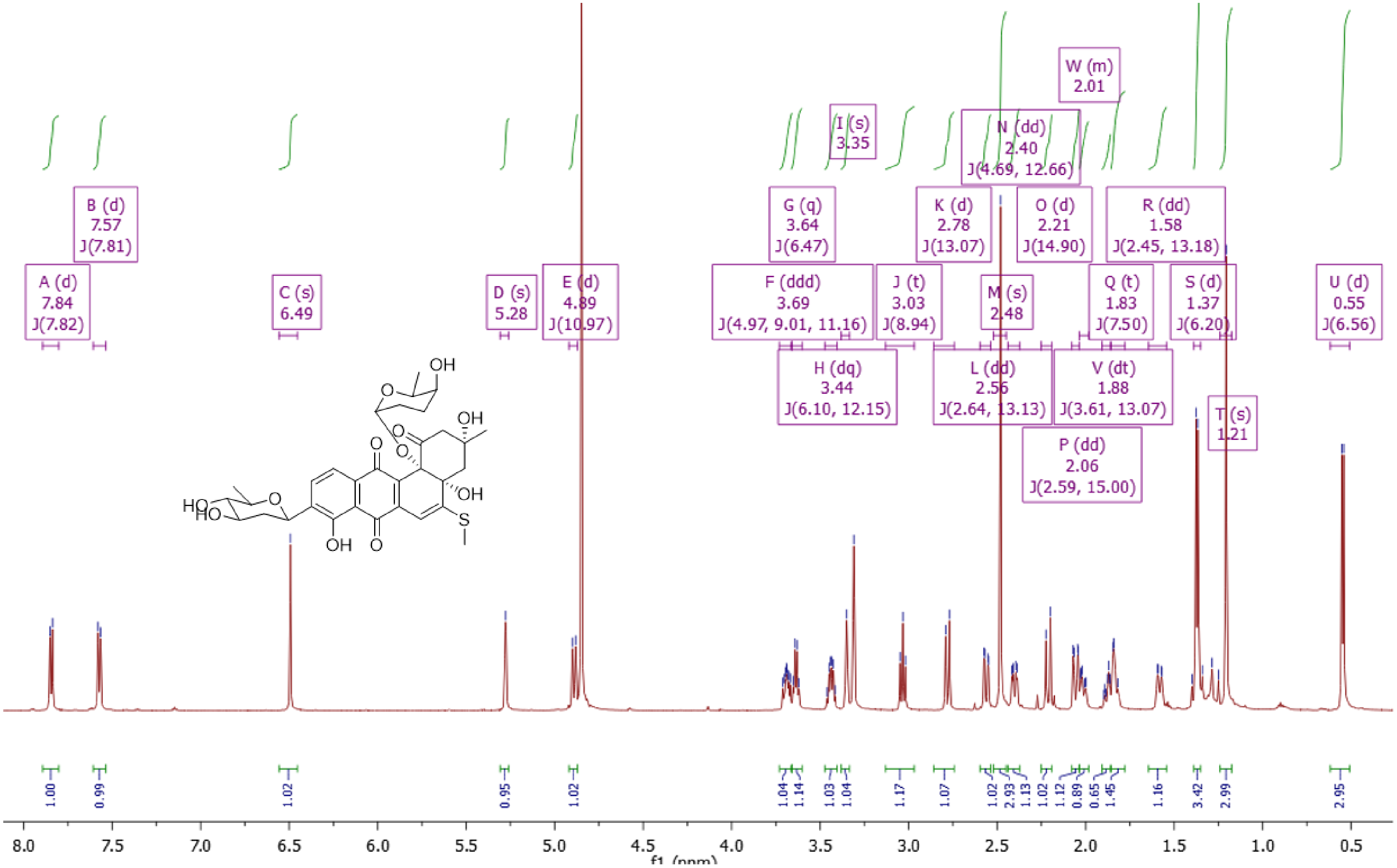

Figure S9. ^{1}H NMR spectrum of **2**.

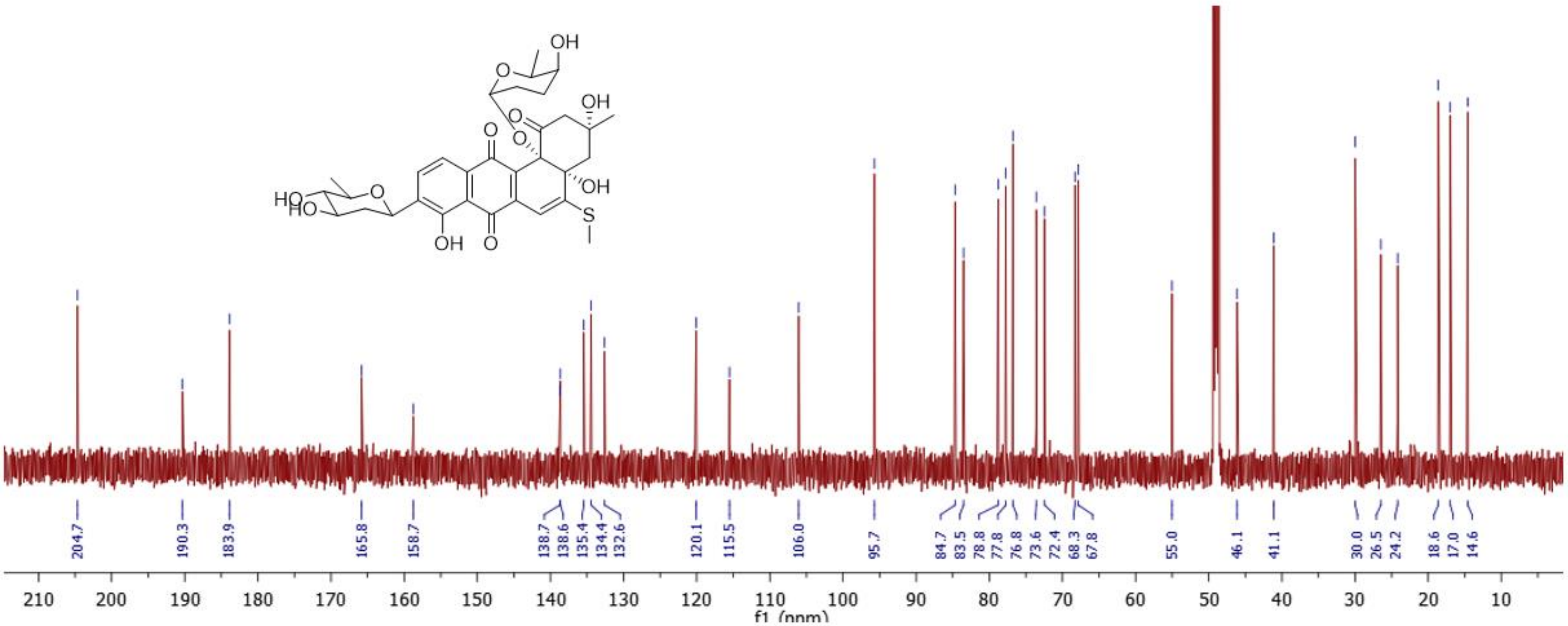

Figure S10. ^{13}C NMR spectrum of **2**.

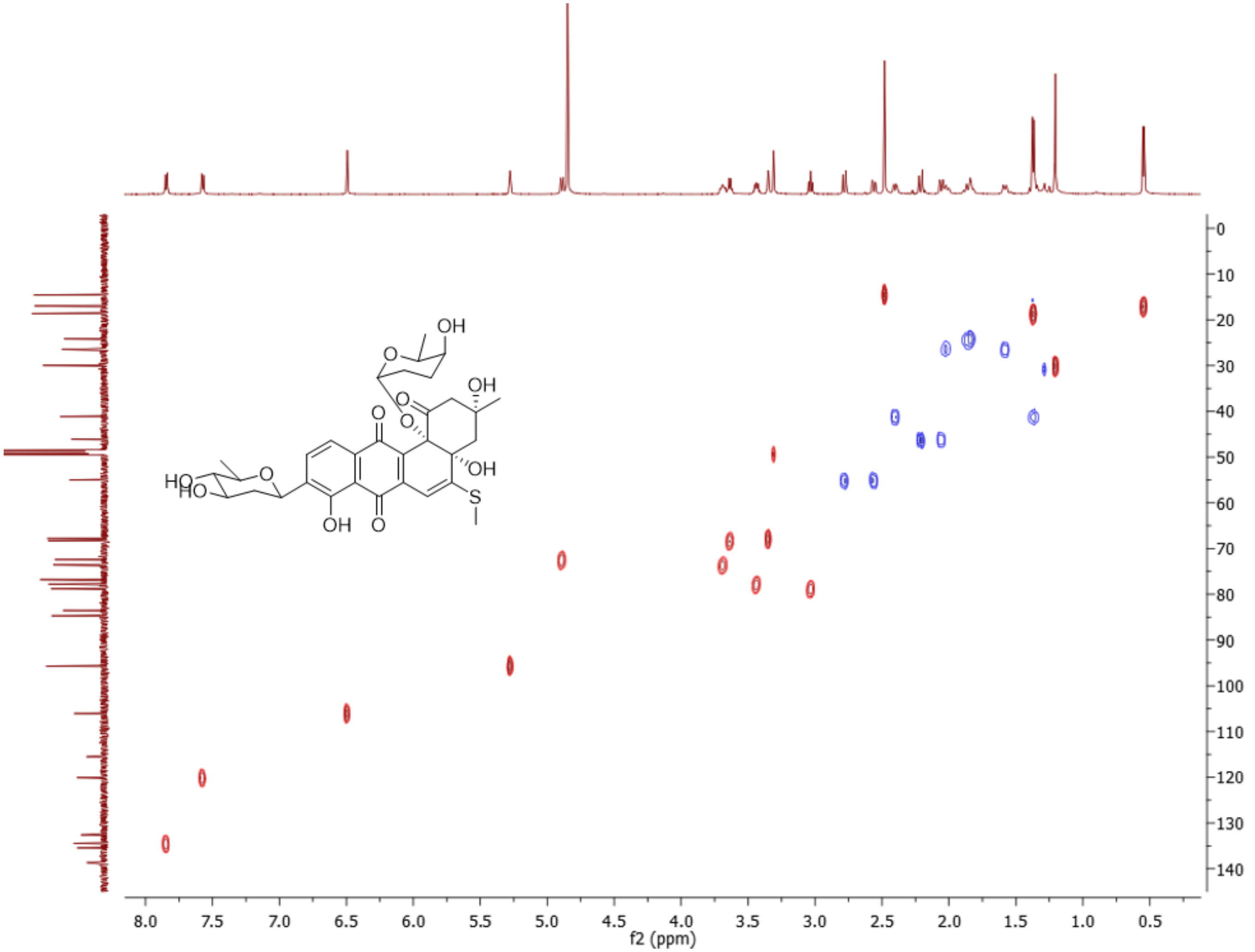

Figure S11. HSQC spectrum of **2**.

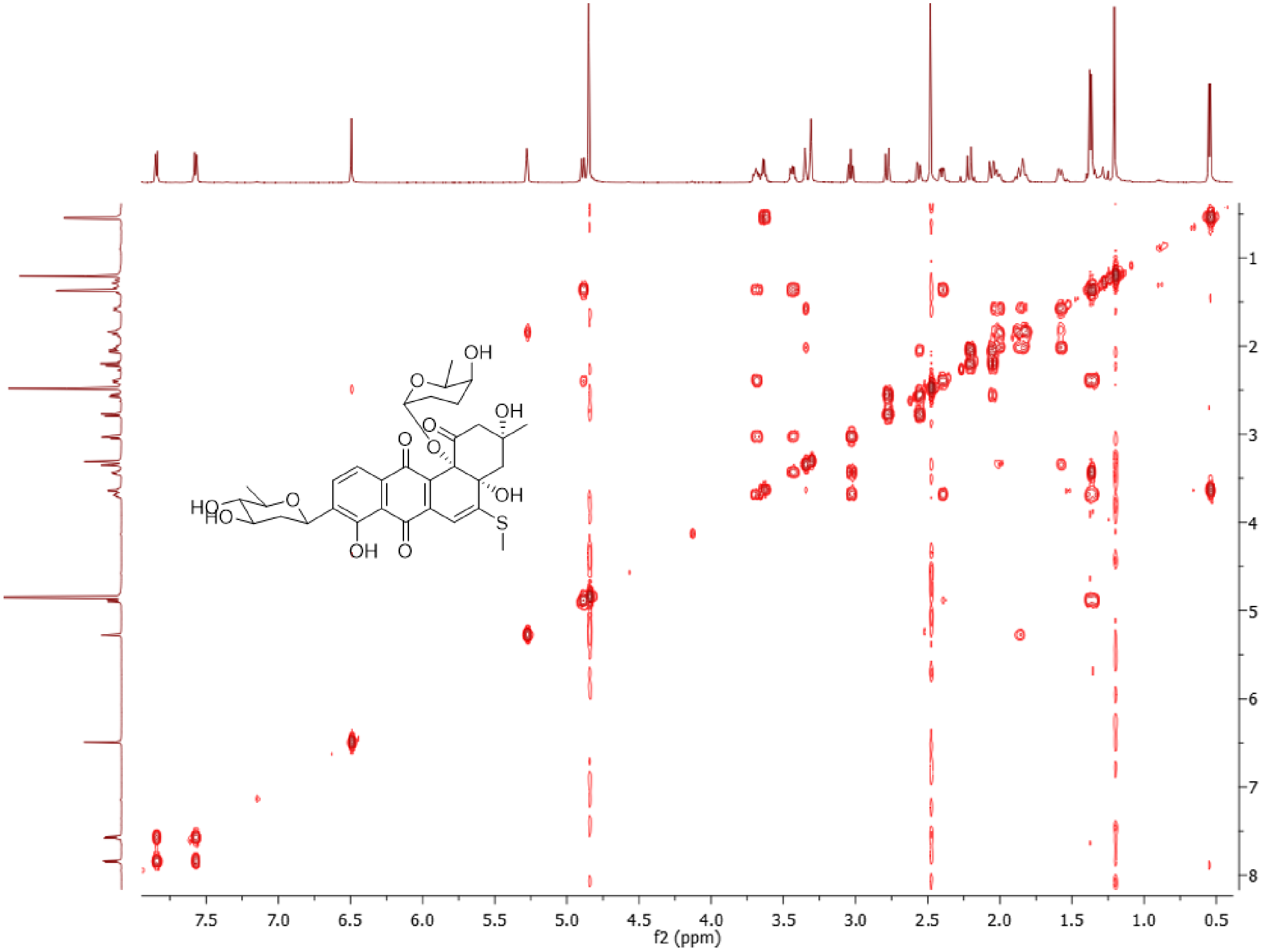

Figure S12. ^{1}H-^{1}H COSY spectrum of **2**.

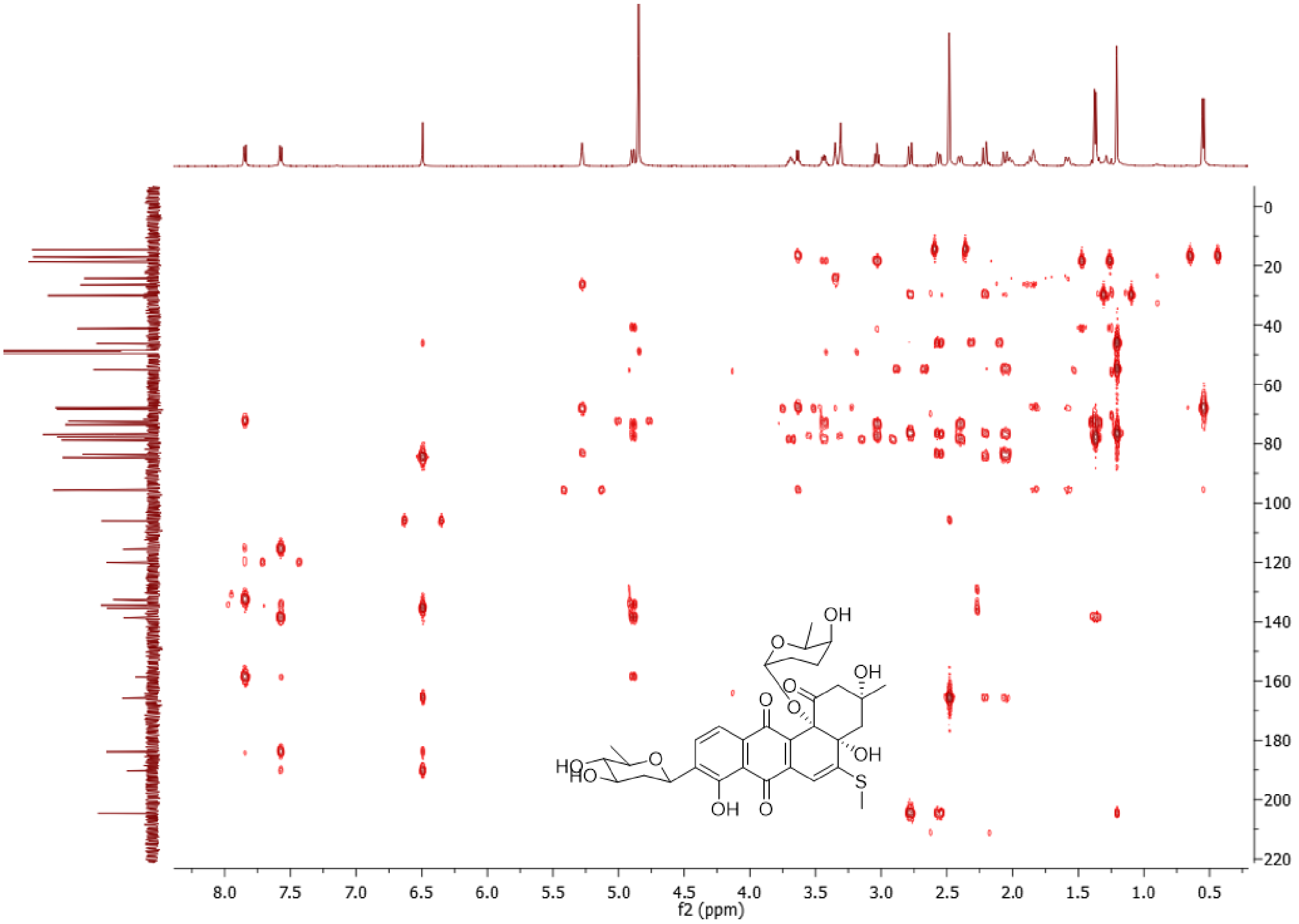

Figure S13. HMBC spectrum of **2**.

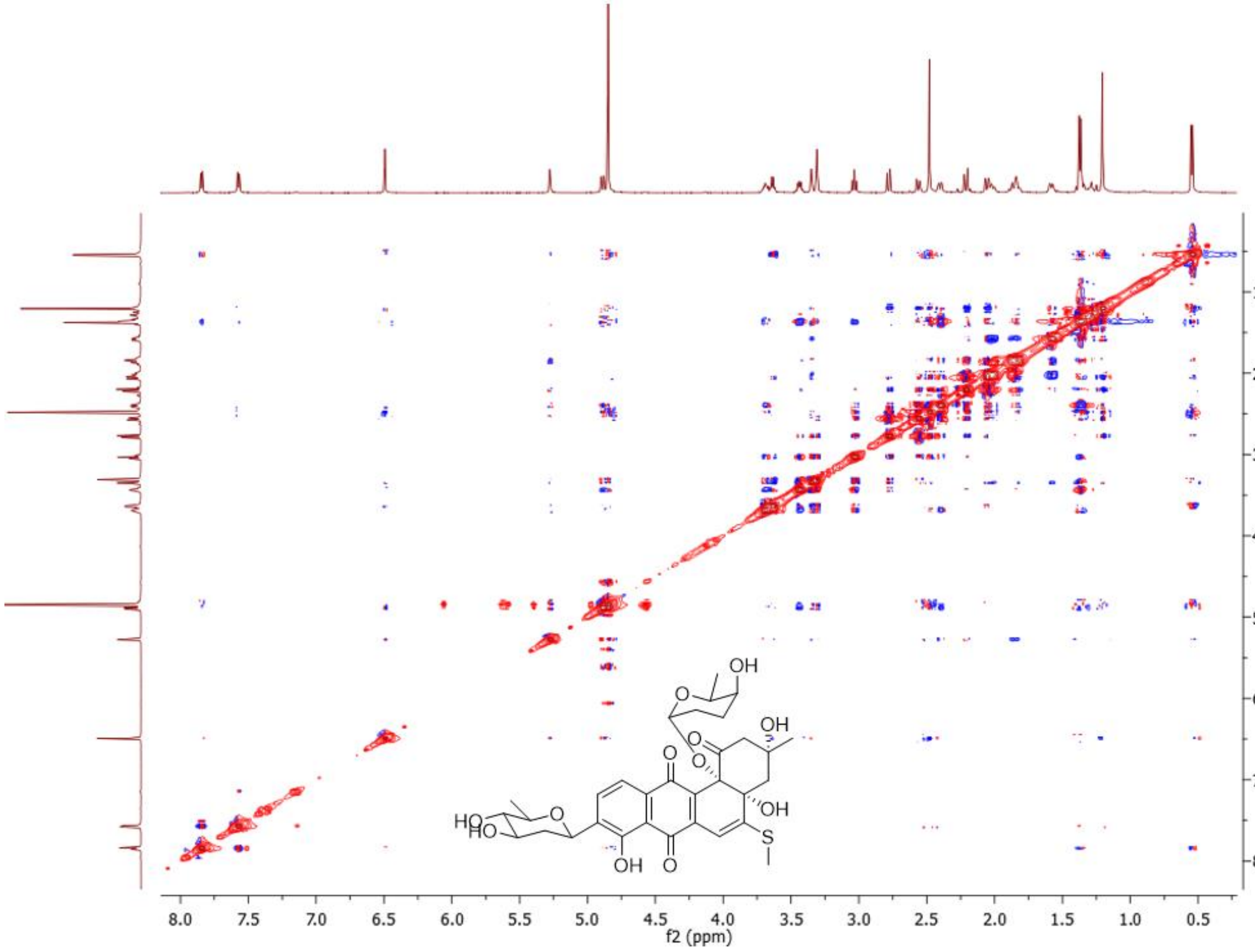

Figure S14. NOESY spectrum of **2**.

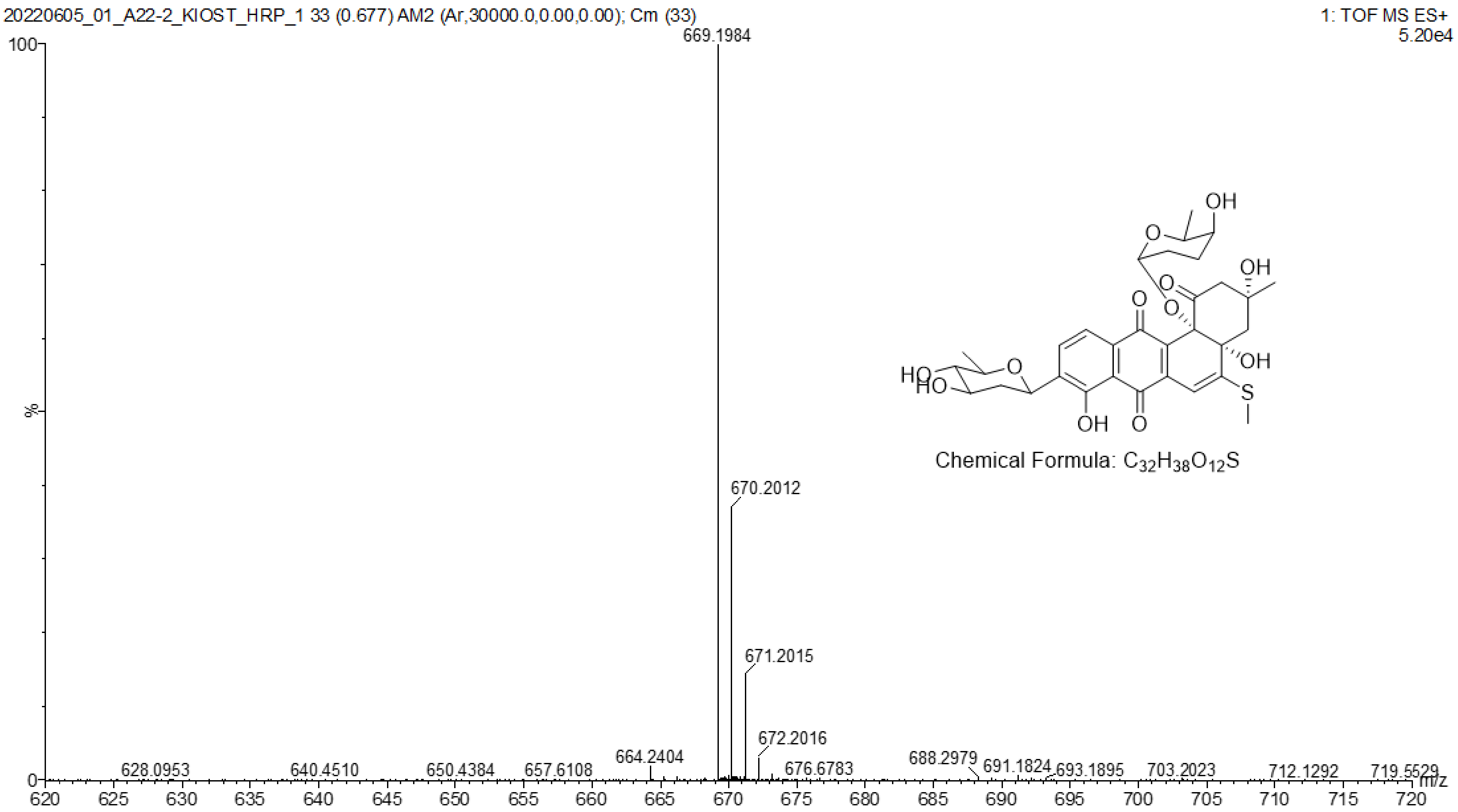

Mass	Calc. Mass	mDa	PPM	DBE	i-FIT	Norm	Conf(%)	Formula
669.1984	669.1982	0.2	0.3	13.5	508.3	0.415	66.05	C32 H38 O12 S Na
	669.1972	1.2	1.8	21.5	518.0	10.156	0.00	C37 H33 O12
	669.2006	-2.2	-3.3	16.5	509.0	1.080	33.95	C34 H37 O12 S

Figure S15. HRESIMS data of **2**.

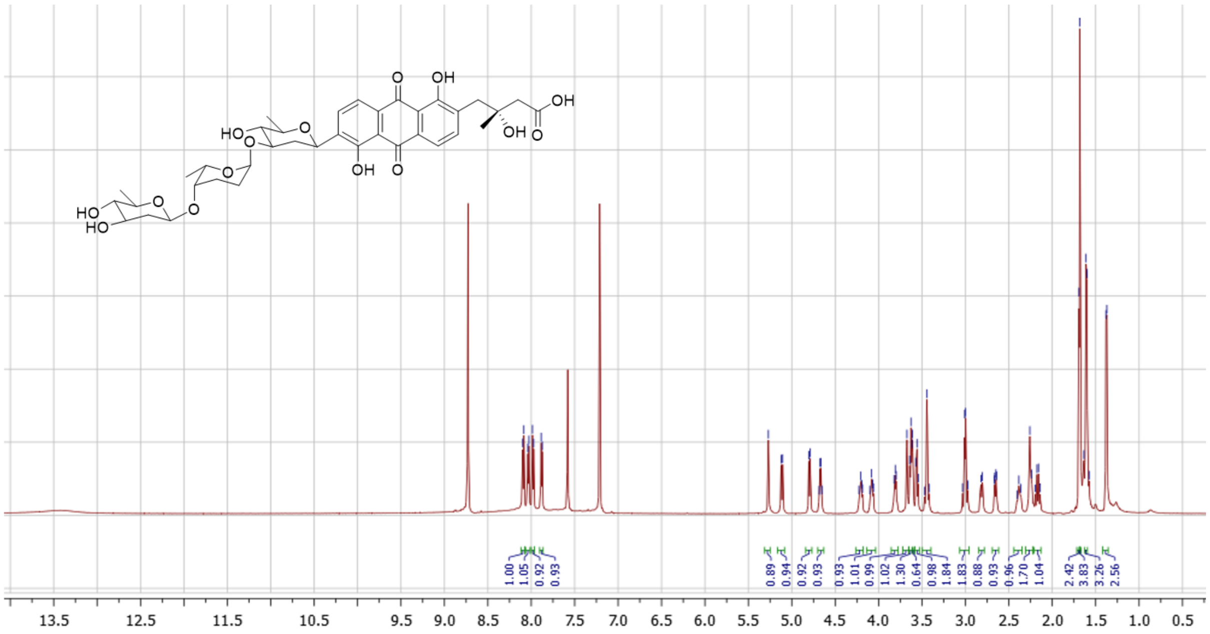

Figure S16. ^{1}H NMR spectrum of **9** (pyridine-d_5).

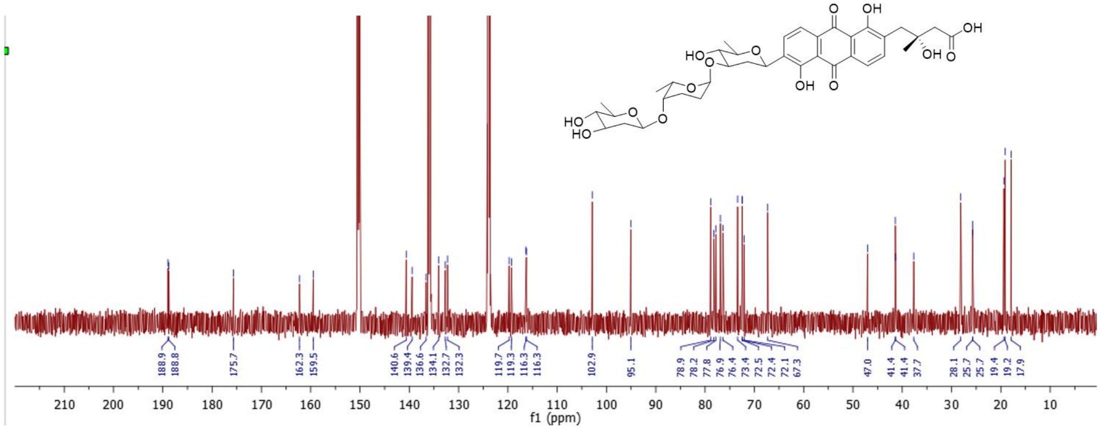

Figure S17. ^{13}C NMR spectrum of **9** (pyridine-d_5).

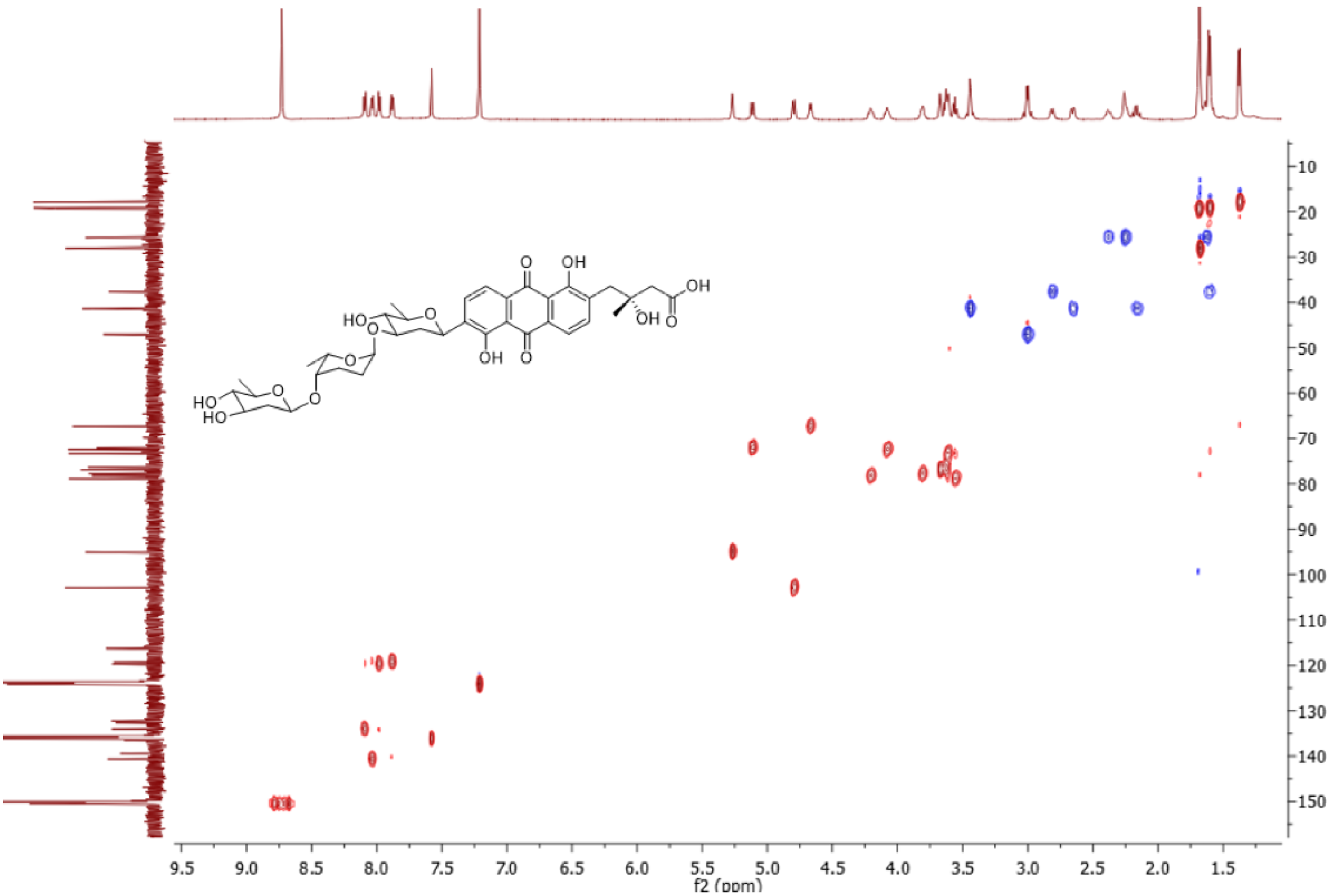

Figure S18. HSQC spectrum of **9** (pyridine-d_5).

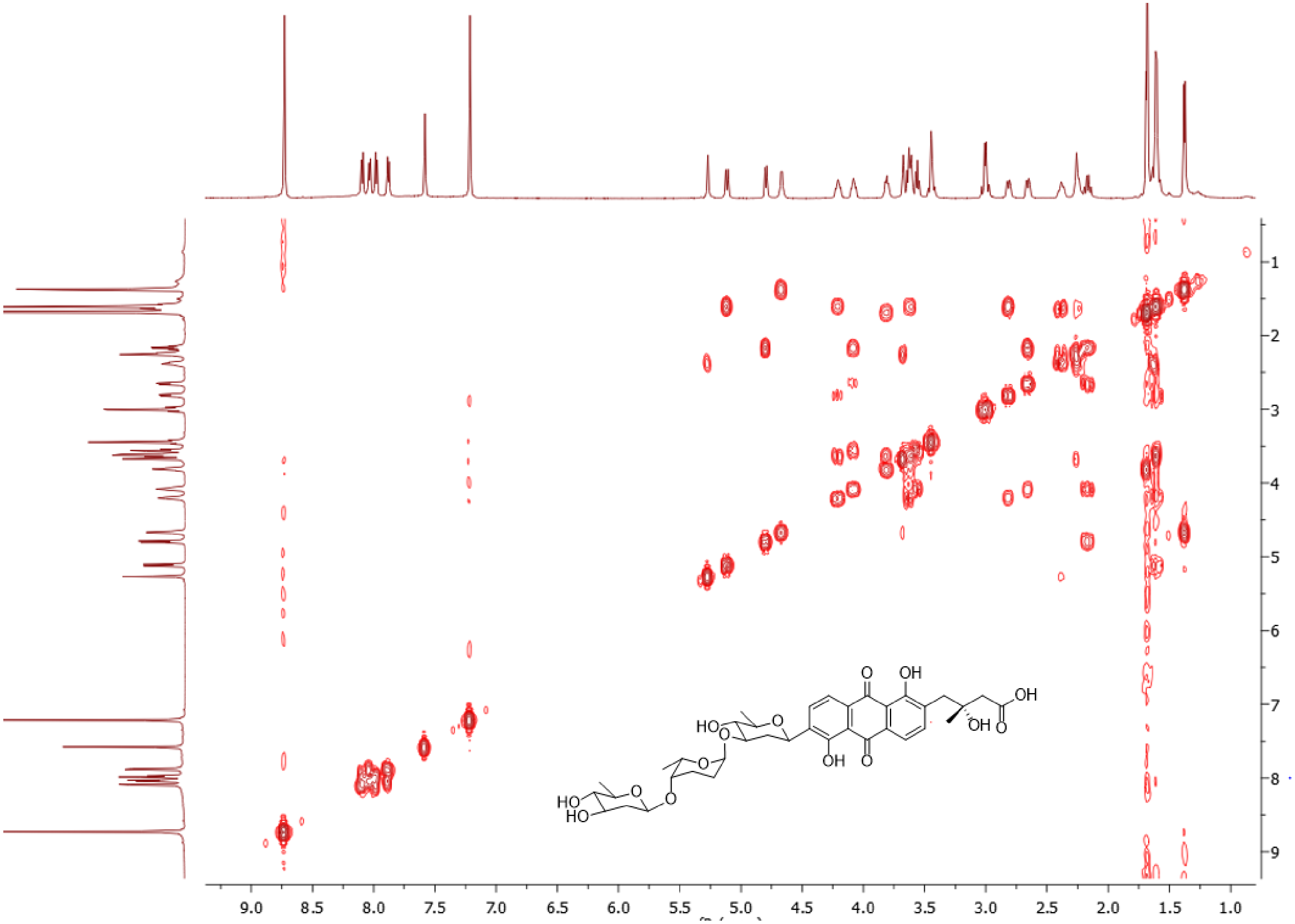

Figure S19. ^{1}H-^{1}H COSY spectrum of **9** (pyridine-d_5).

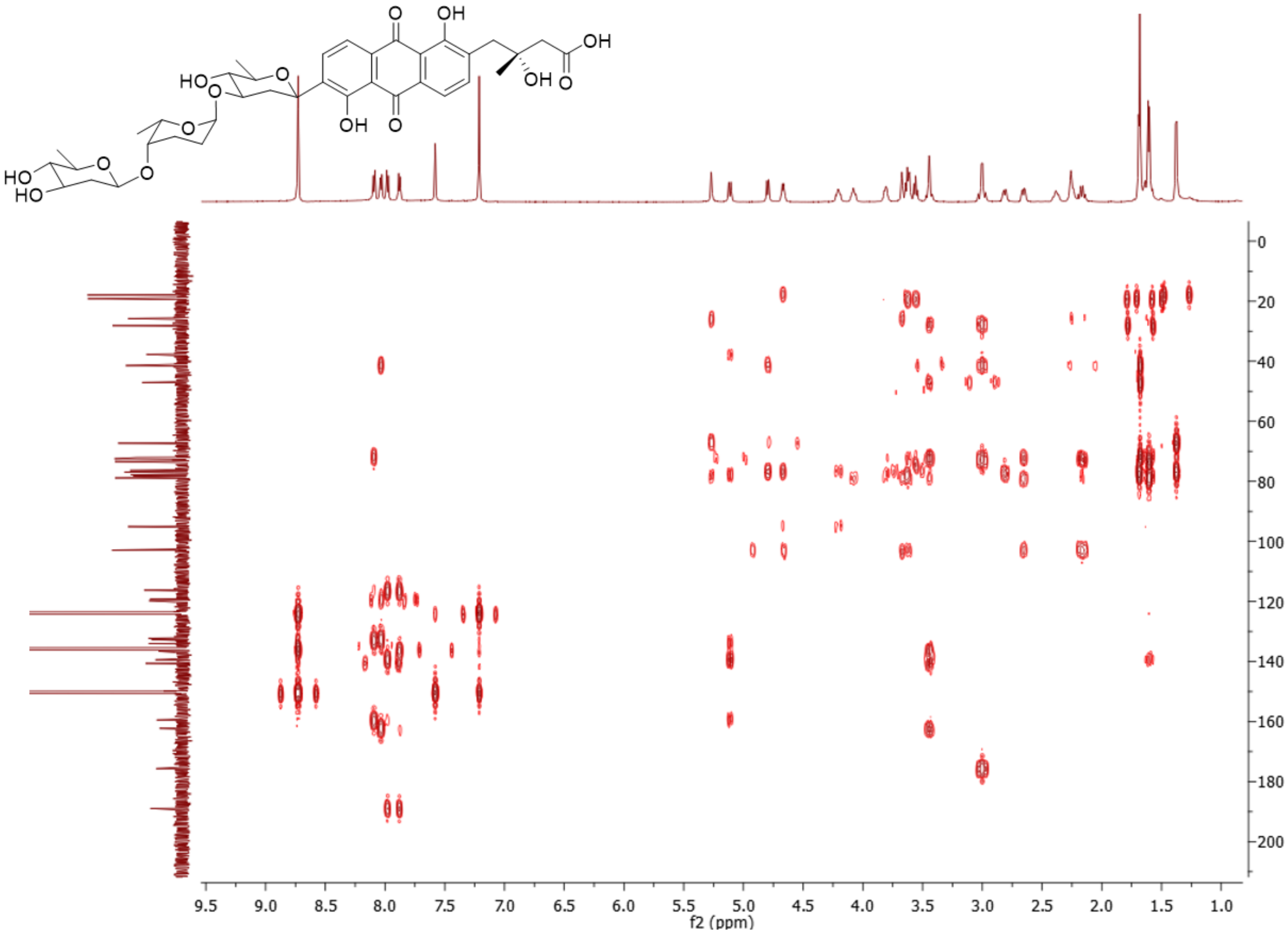

Figure S20. HMBC spectrum of **9** (pyridine-d_5).

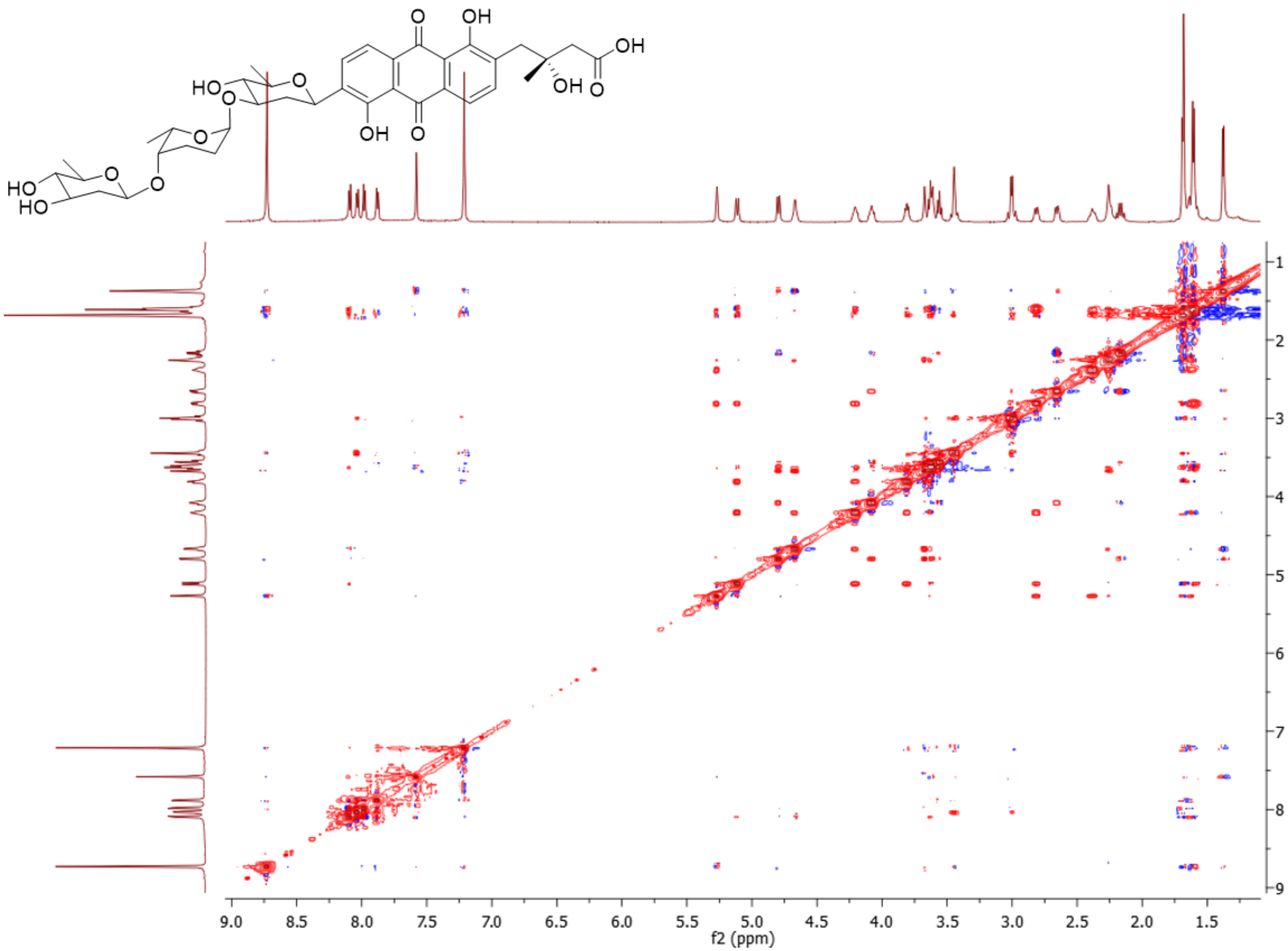

Figure S21. NOESY spectrum of **9** (pyridine-d_5).

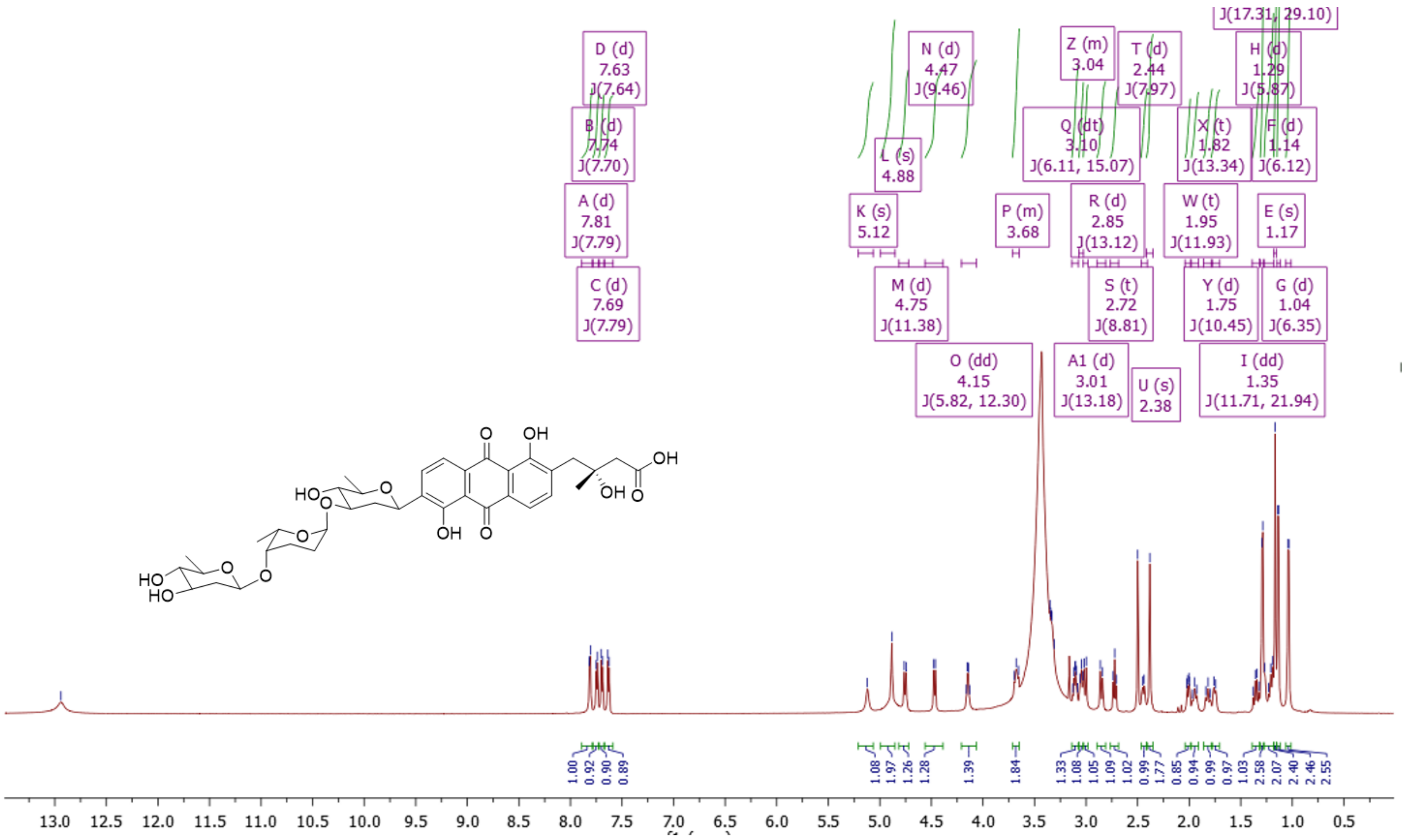

Figure S22. ^{1}H NMR spectrum of **9** (DMSO-d_6).

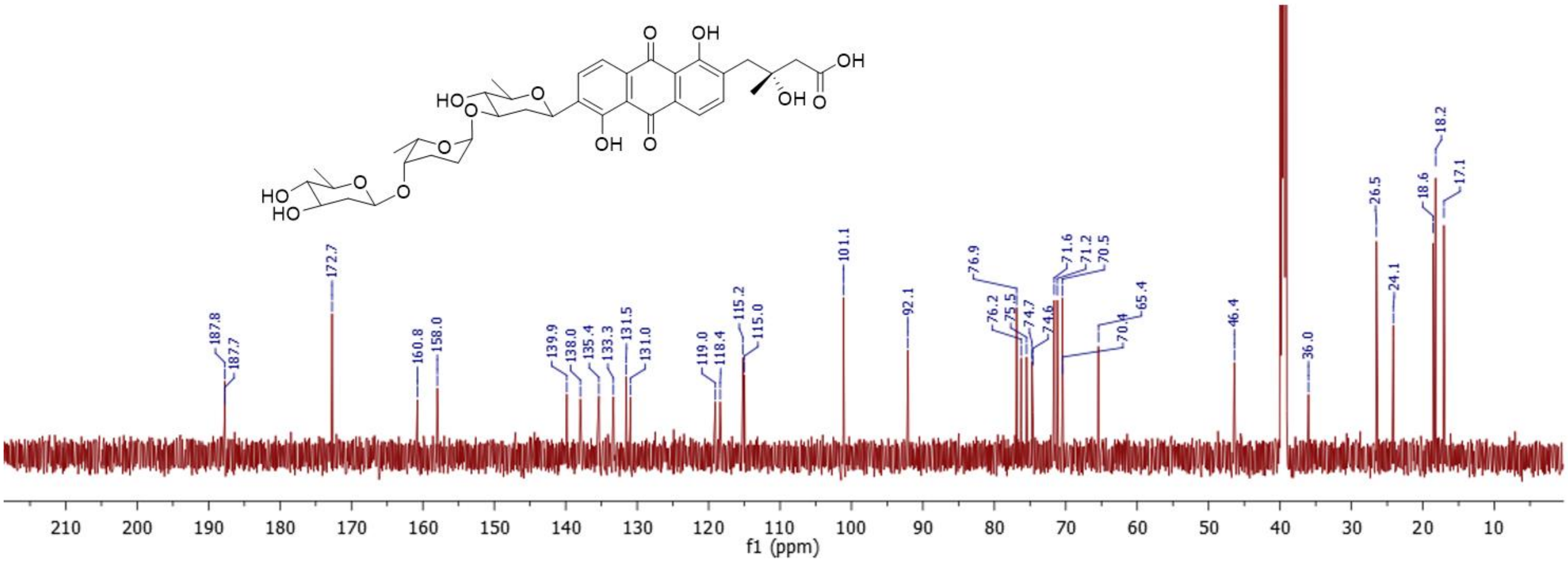

Figure S23. ^{13}C NMR spectrum of **9** (DMSO-d_6).

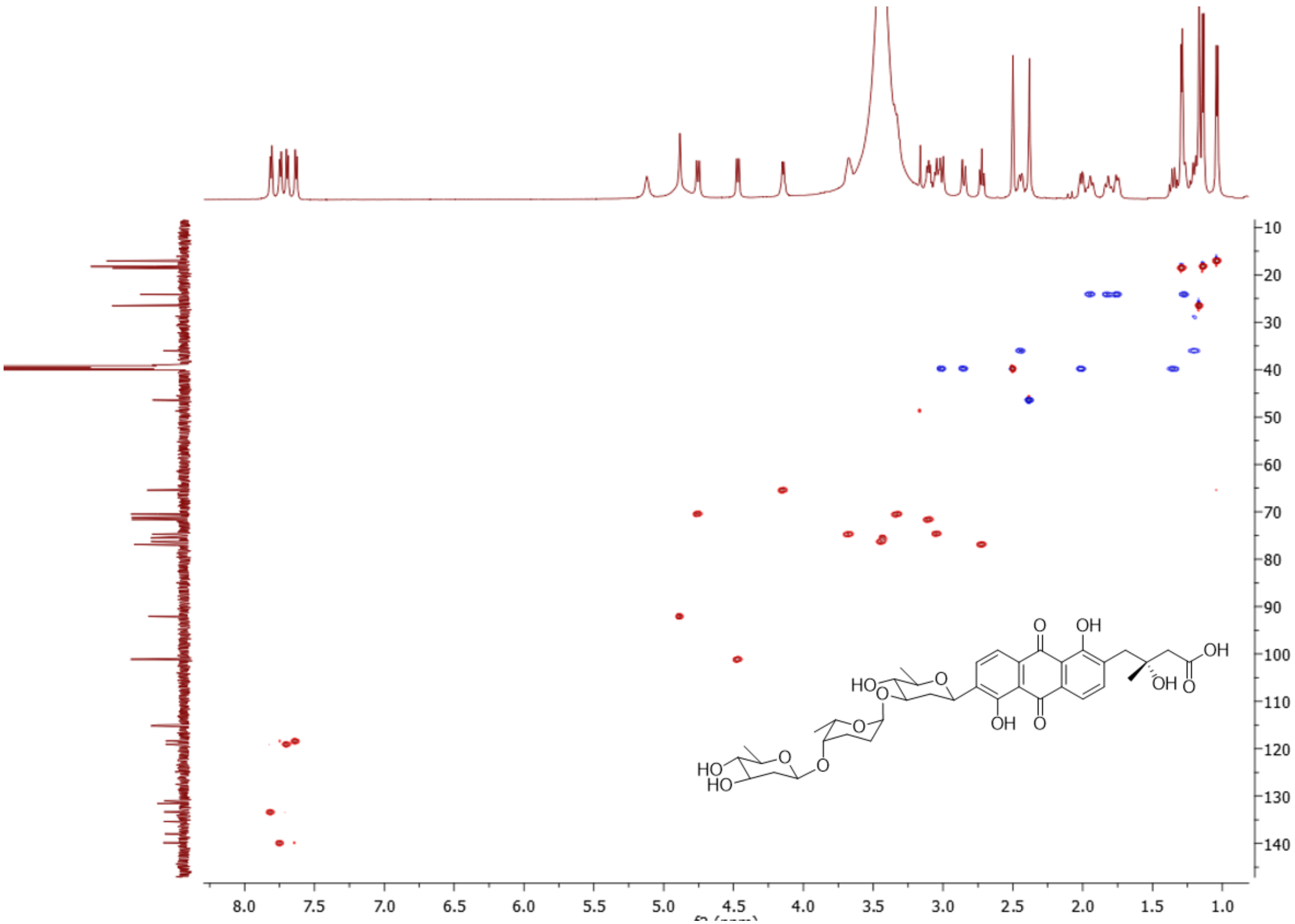

Figure S24. HSQC spectrum of **9** (DMSO-d_6).

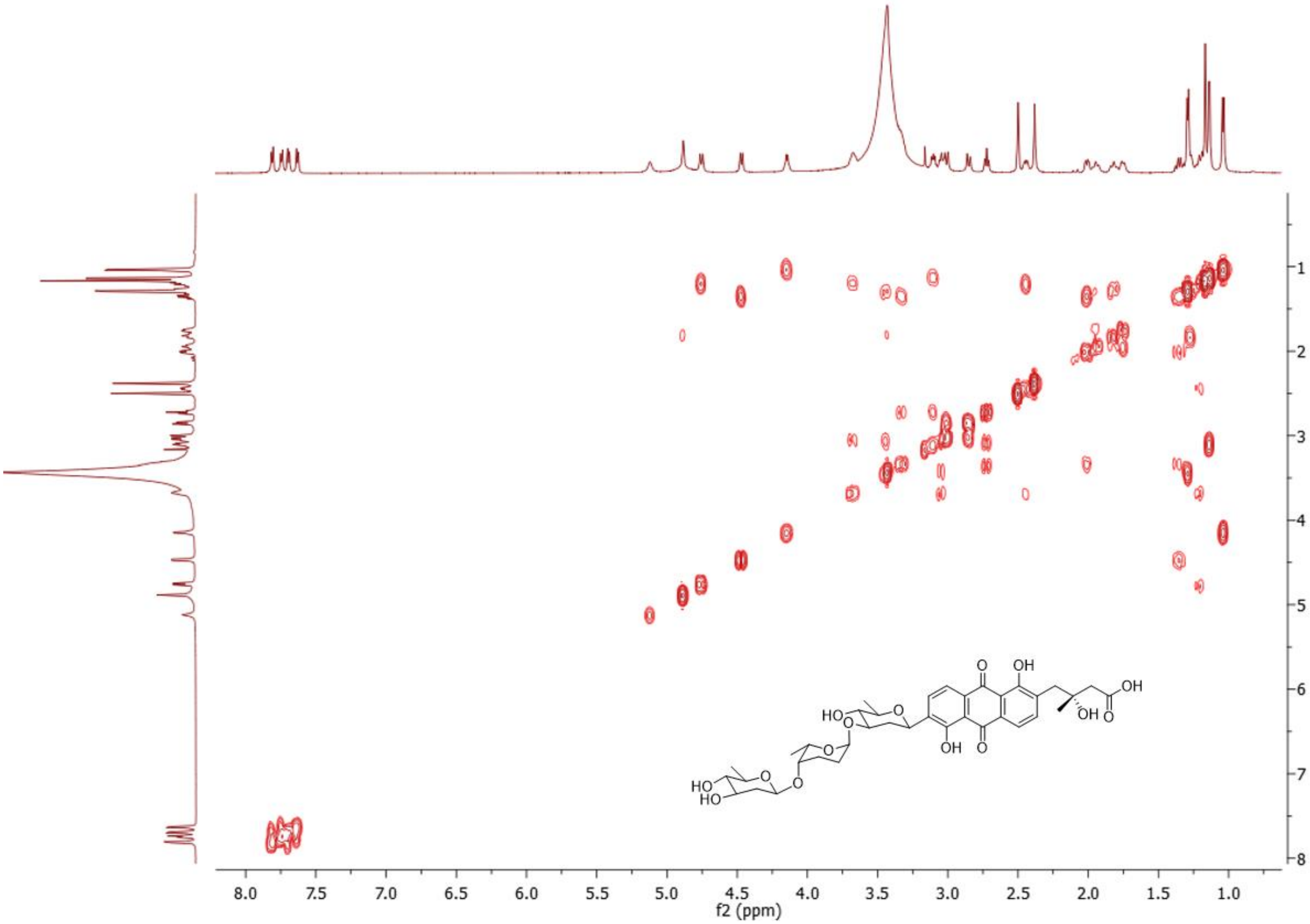

Figure S25. ^{1}H-^{1}H COSY spectrum of **9** (DMSO-d_6).

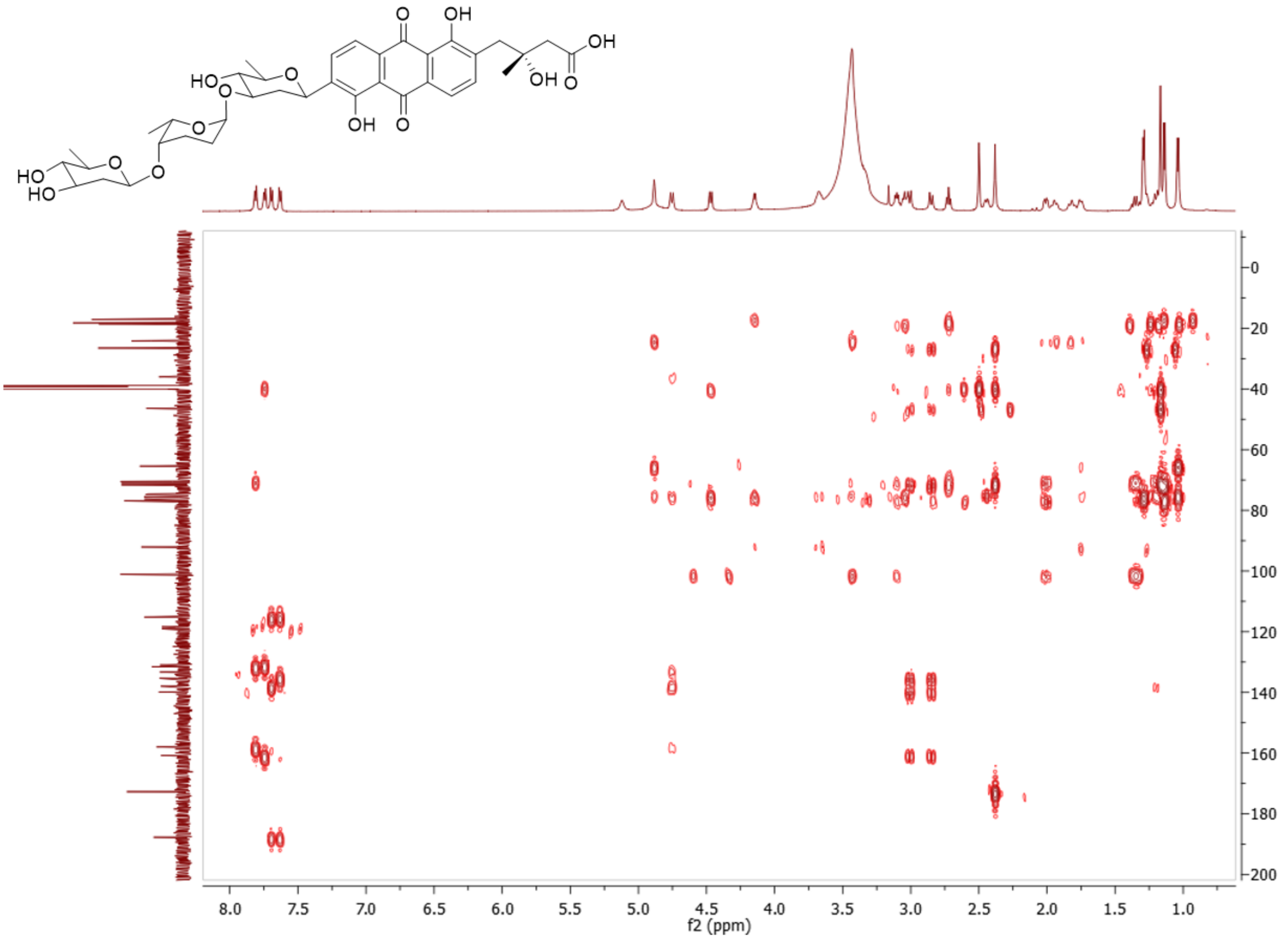

Figure S26. HMBC spectrum of **9** (DMSO-d_6).

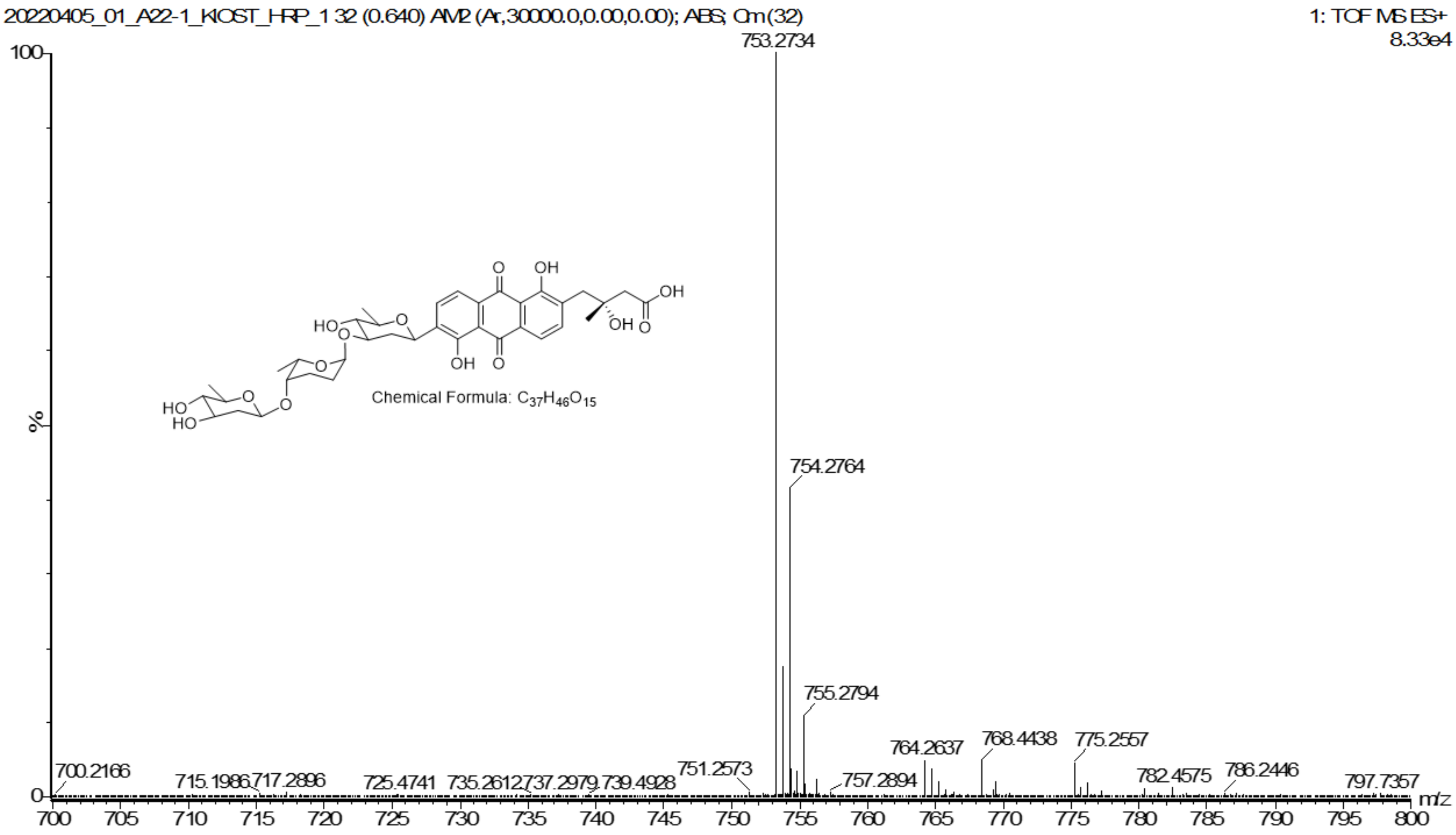

Mass	Calc. Mass	mDa	PPM	DBE	i-FIT	Norm	Conf(%)	Formula
753.2734	753.2734	0.0	0.0	14.5	419.4	0.004	99.65	C37 H46 O15 Na
	753.2758	-2.4	-3.2	17.5	425.1	5.651	0.35	C39 H45 O15

Figure S27. HRESIMS data of **9**.

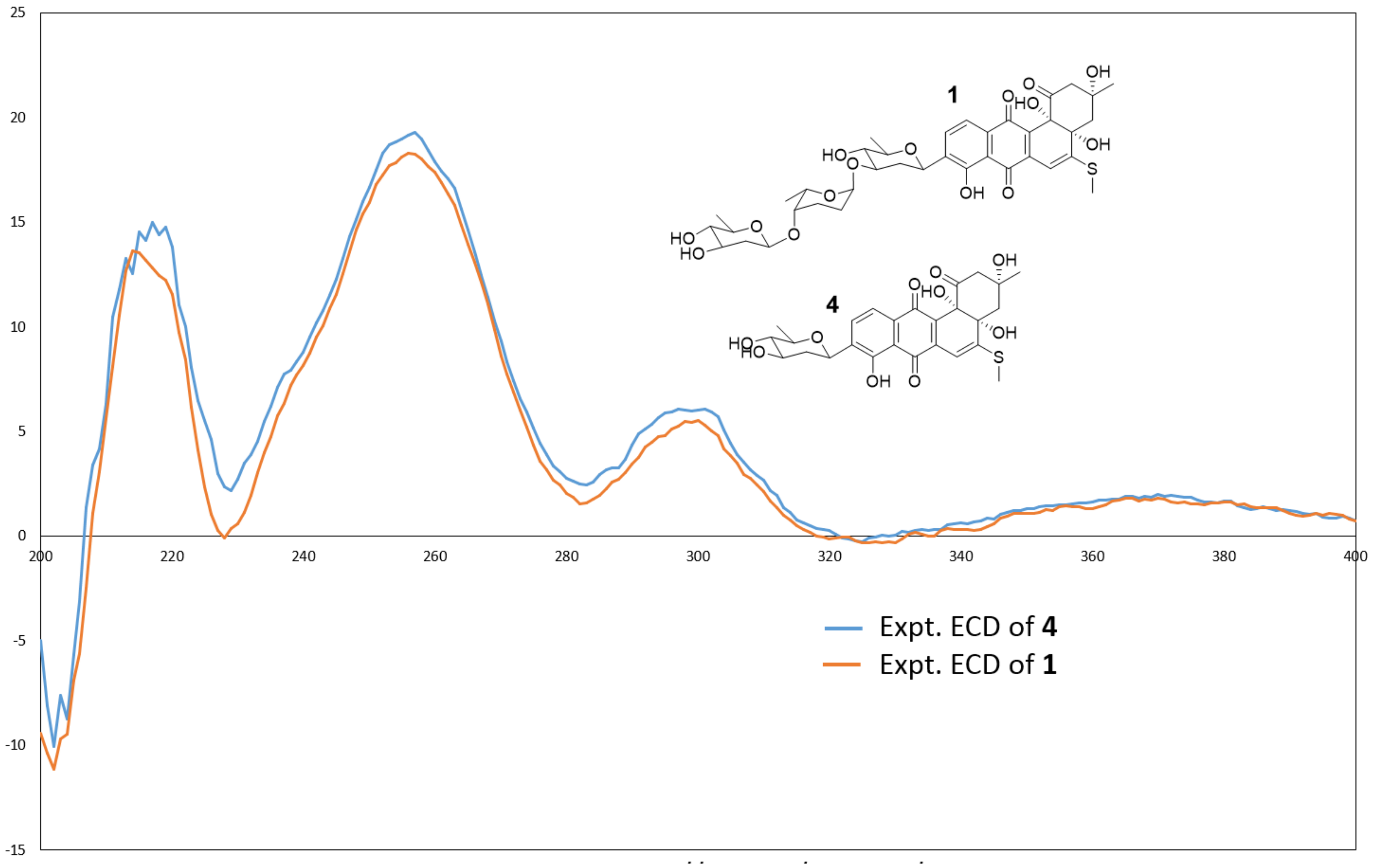

Figure S28. Comparison of ECD spectra between **1** and **4**.

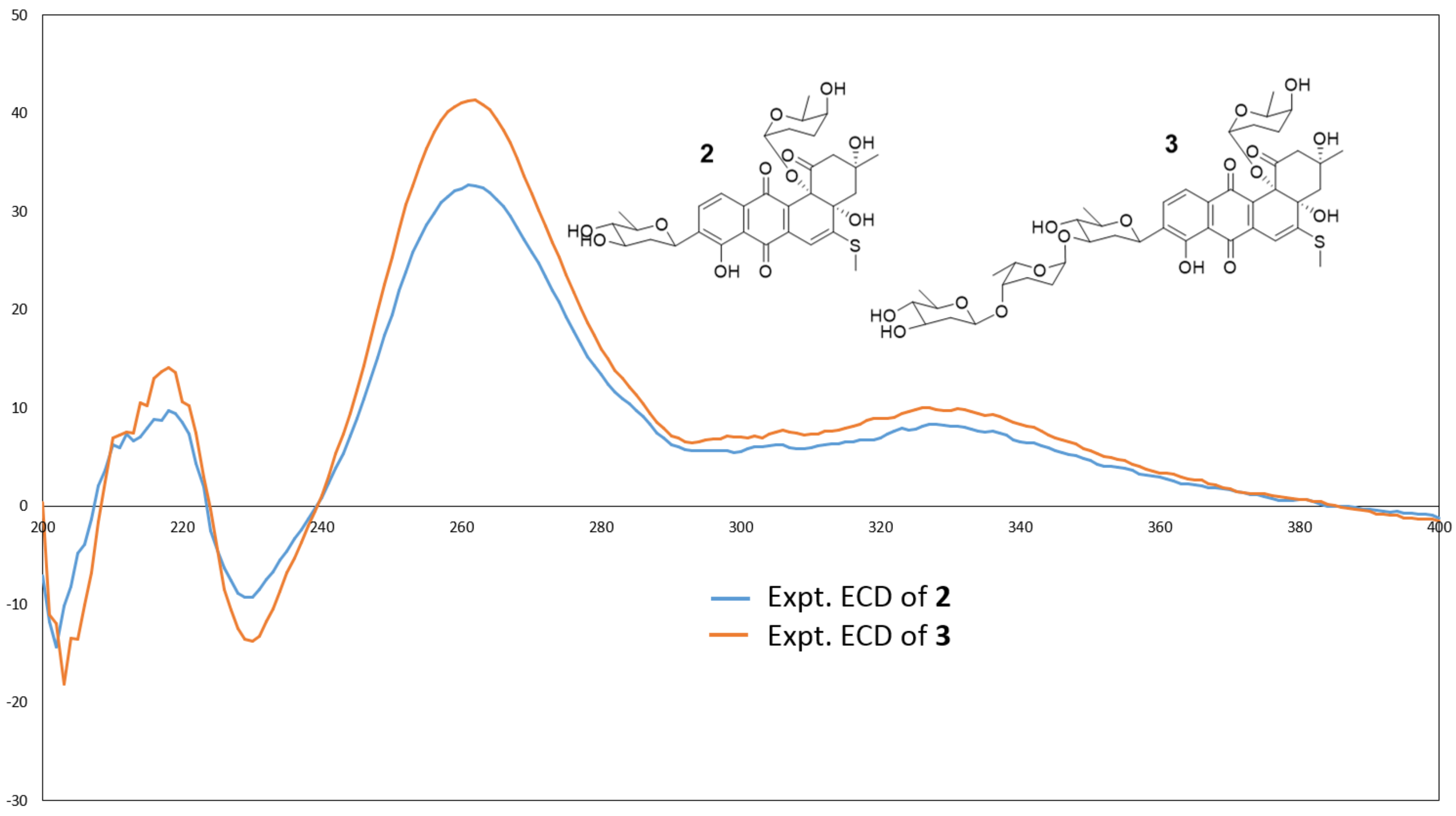

Figure S29. Comparison of ECD spectra between **2** and **3**.

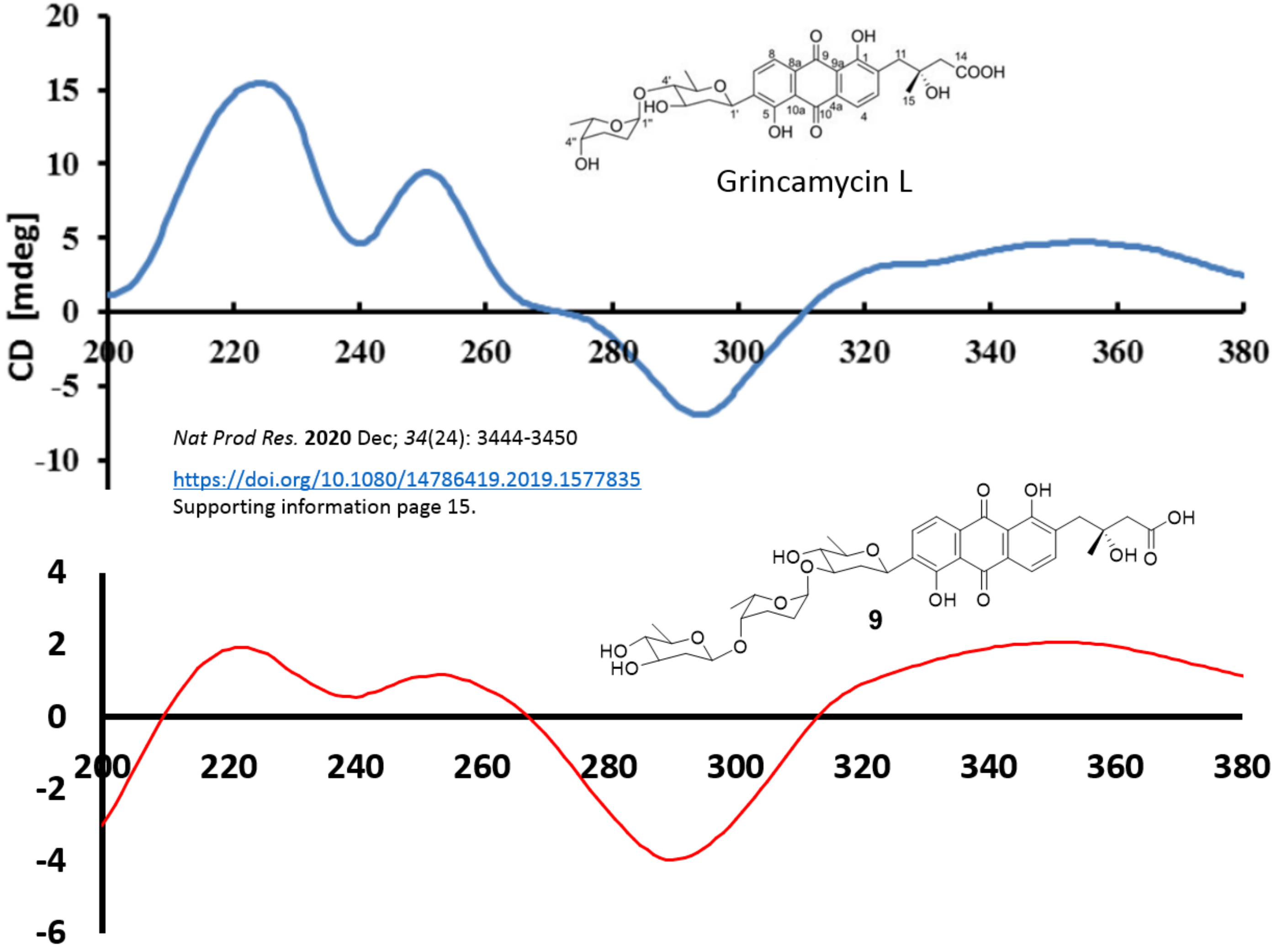

Figure S30. Comparison of ECD spectra between grincamycin U (**9**) and grincamycin L.

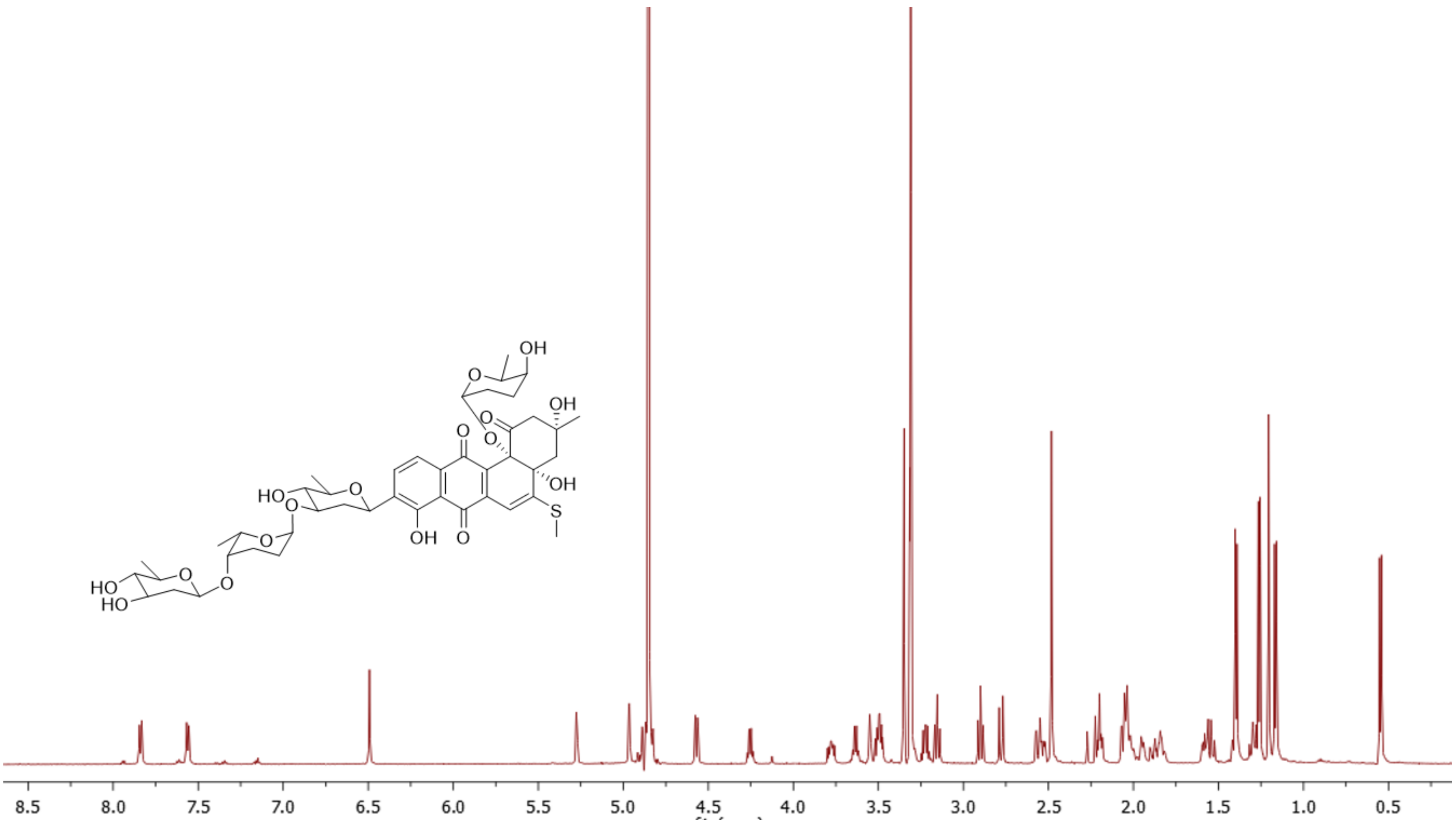

Figure S31. ^{1}H NMR spectrum of **3**.

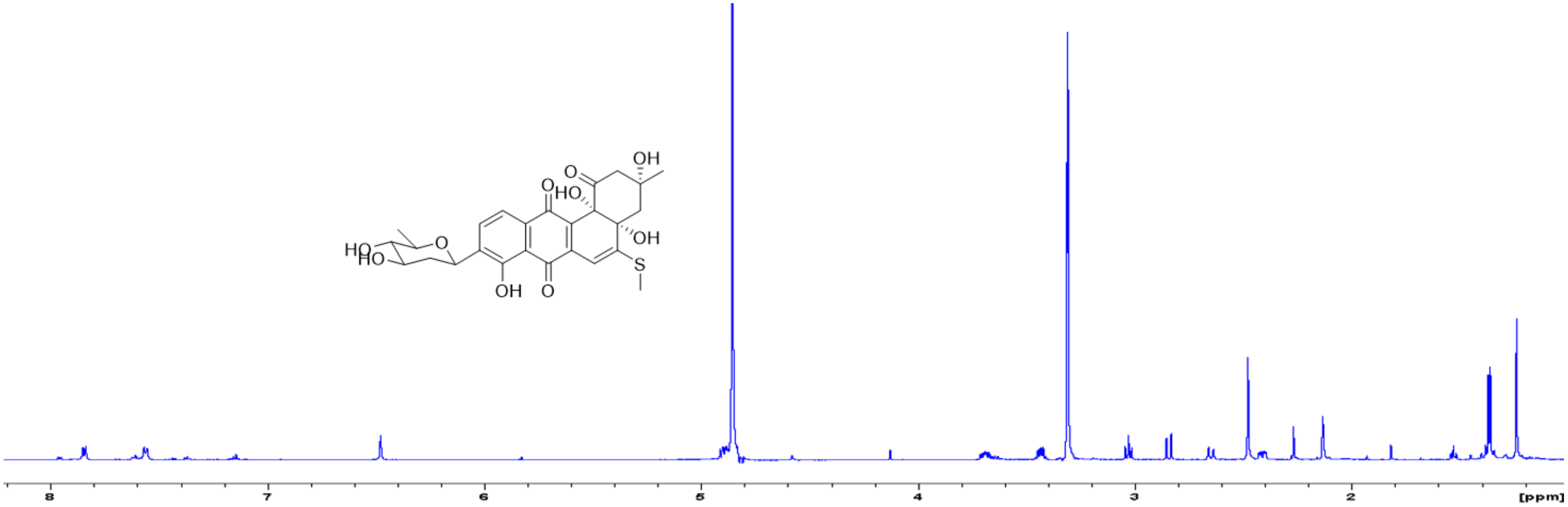

Figure S32. ^{1}H NMR spectrum of **4**.

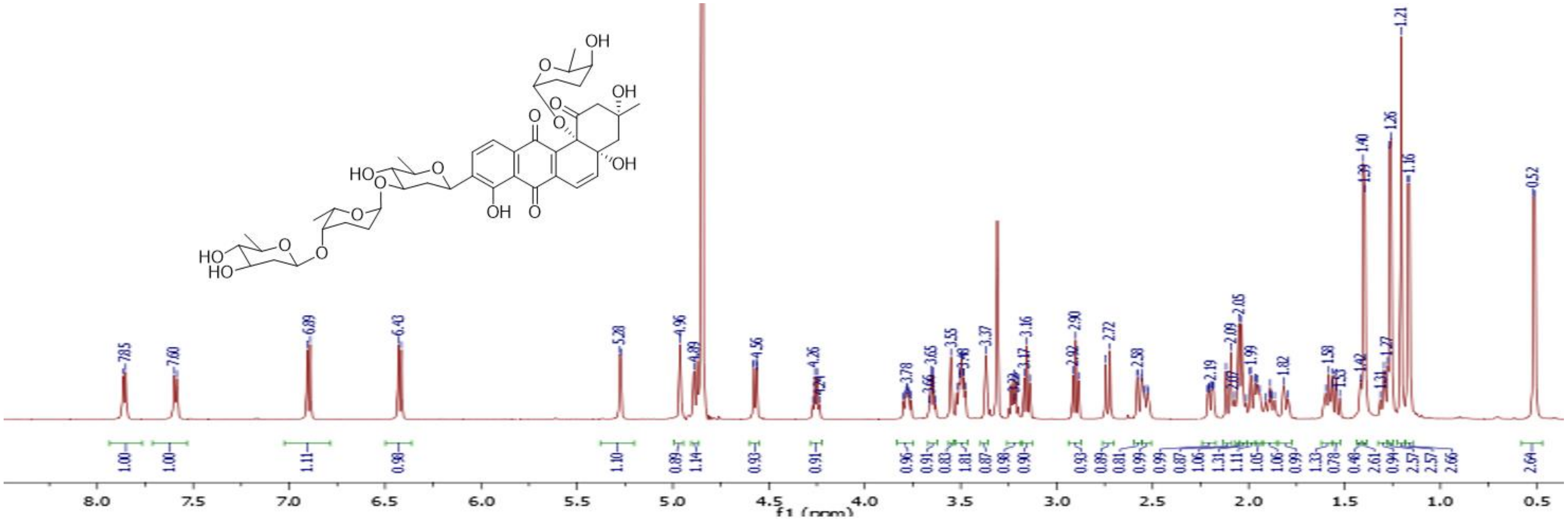

Figure S33. ^{1}H NMR spectrum of **5**.

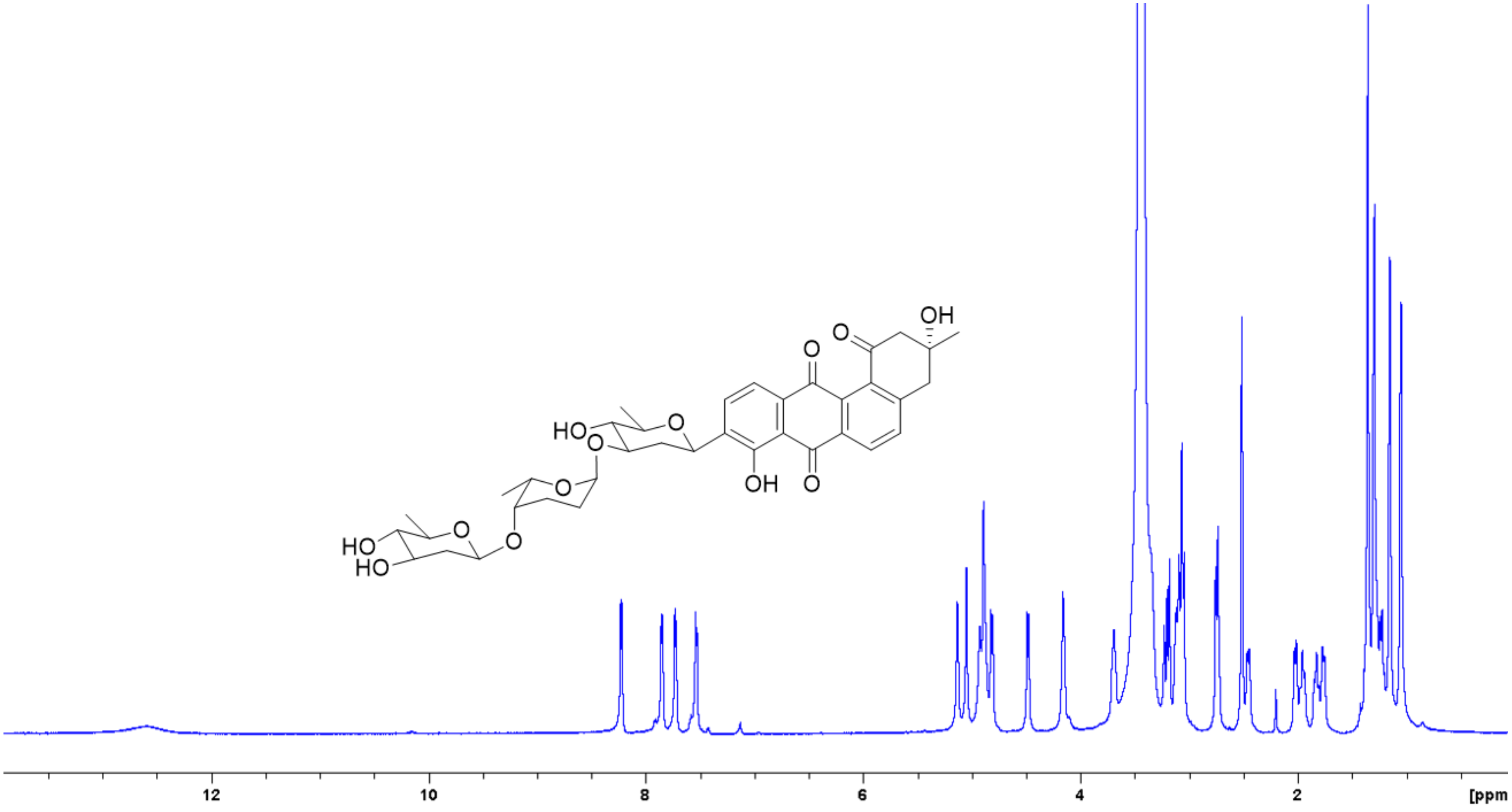

Figure S34. ^{1}H NMR spectrum of **6**.

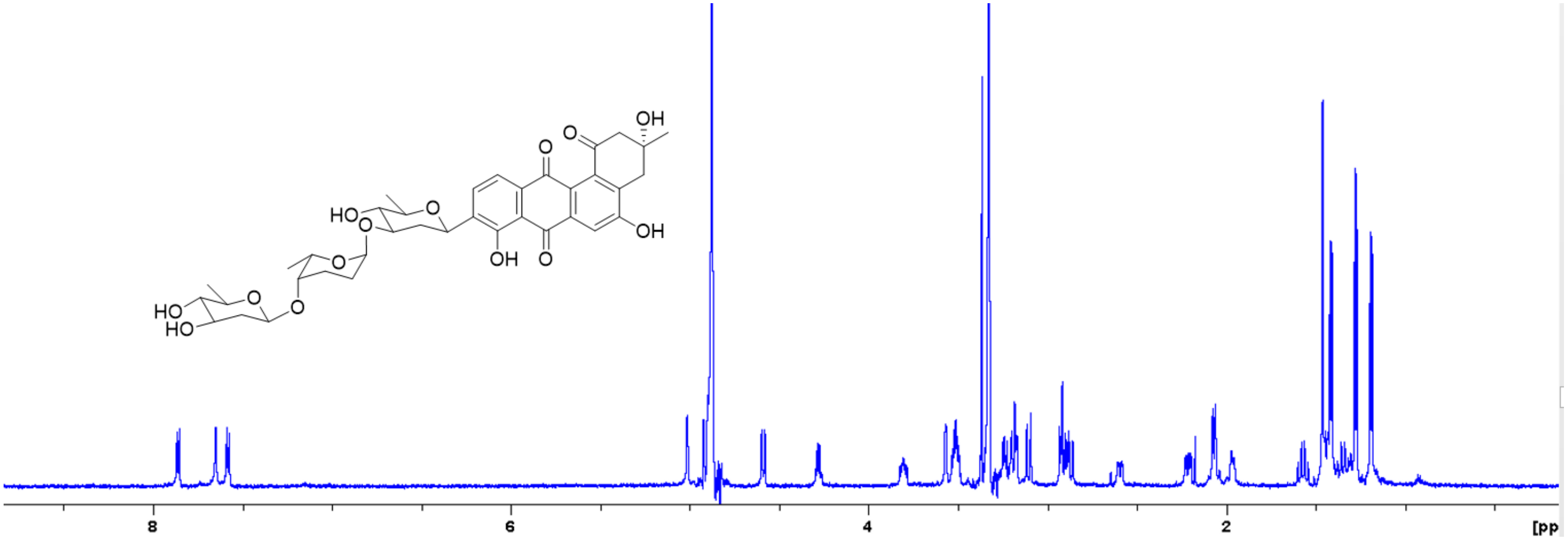

Figure S35. ^{1}H NMR spectrum of **7**.

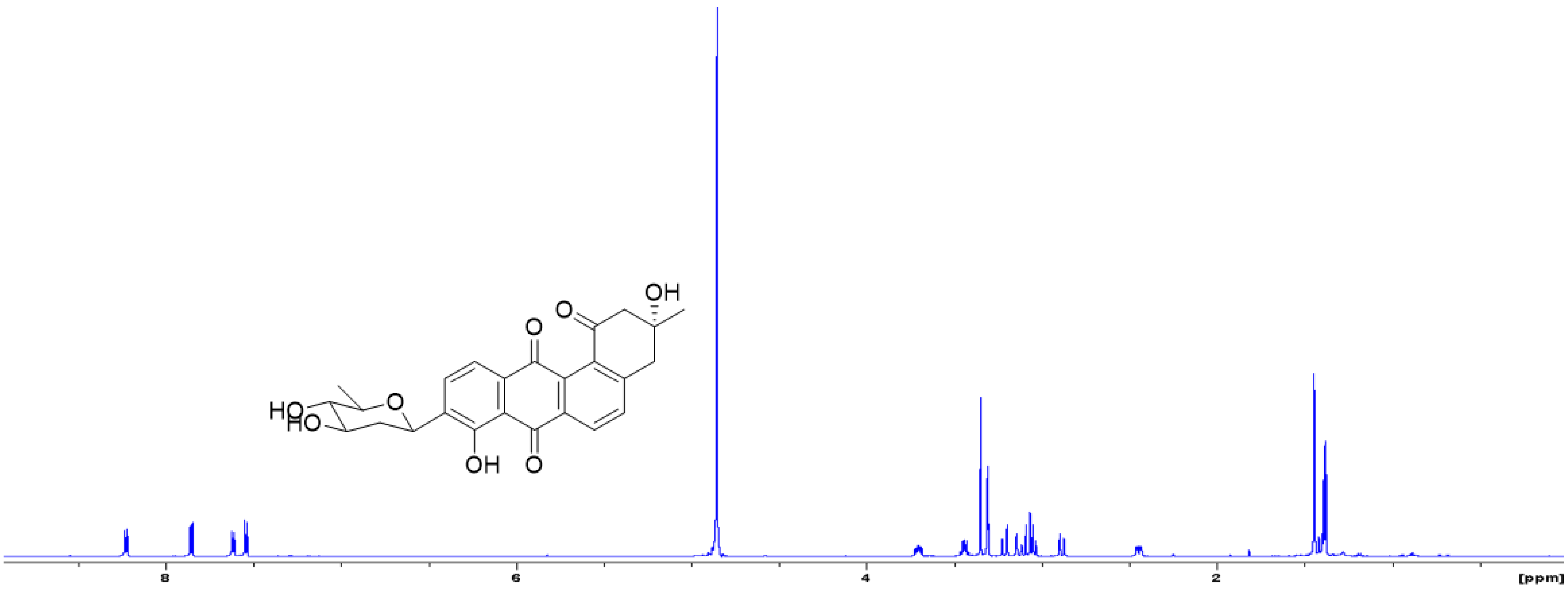

Figure S36. ^{1}H NMR spectrum of **8**.

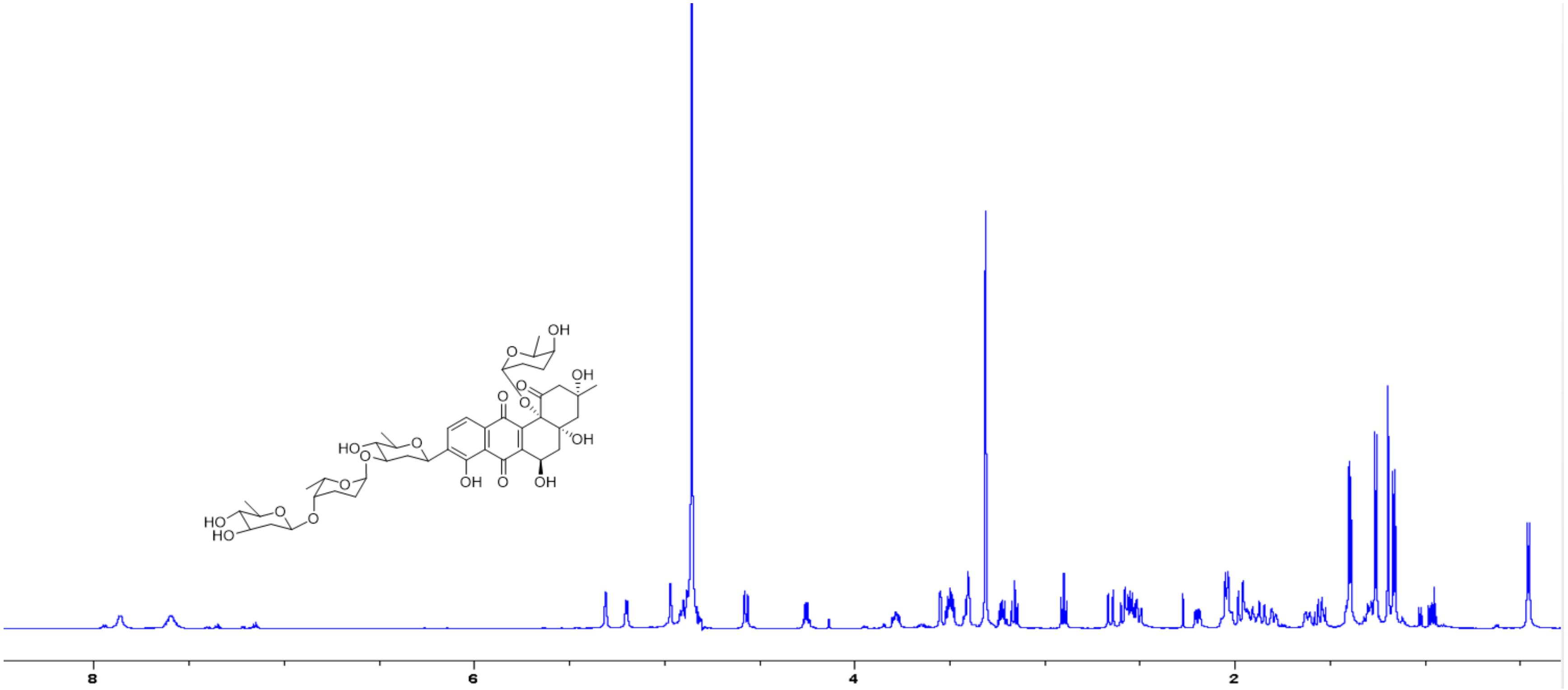

Figure S37. ^{1}H NMR spectrum of **10**.

ACHN

	1		2			3	
Conc.(ug/	Mean	SD	Mean	SD	Conc.(ug/	Mean	SD
0.3	21.33862	8.37351	12.91978	5.055993	0.3	16.74888	5.155391
0.1	53.87127	5.715222	32.78918	4.718279	0.1	45.73991	4.644665
0.03	68.54012	3.900378	72.29478	1.321422	0.03	57.26457	4.503098
0.01	77.47202	9.3908	89.94869	2.657495	0.01	76.00897	5.705476
0.003	83.04572	5.117508	92.35075	5.585086	0.003	90.53811	6.131321
0.001	97.97108	2.511589	100.3032	3.929275	0.001	99.59641	7.098806
GI50	0.081	0.009	0.060	0.003	GI50	0.054	0.000
GI50 (μM)	0.104	0.012	0.093	0.004		0.060	0.001

	9			Adriamycin	
Conc.(ug/	Mean	SD	Conc.(ug/	Mean	SD
30	-12.1059	3.529526	3	-7.61364	4.142353
10	13.62612	6.849341	1	-3.97727	4.510395
3	41.5259	4.358922	0.3	15.29545	2.959202
1	82.62951	7.421263	0.1	28.93182	3.500788
0.3	97.38176	4.598658	0.03	94.72727	3.827547
GI50	2.501	0.261	GI50	0.076	0.005
GI50 (μM)	3.422	0.357	GI50 (μM)	0.140	0.009

HCT-15

	1		2			3	
Conc.(ug/	Mean	SD	Mean	SD	Conc.(ug/	Mean	SD
0.3	15.12605	2.387636	17.30567	4.344032	0.3	27.99774	8.543784
0.1	43.43488	4.655546	56.22374	7.045587	0.1	55.28846	9.260079
0.03	62.86765	6.263008	78.49265	6.998446	0.03	60.66176	6.094876
0.01	80.09454	9.102767	82.30042	8.094141	0.01	71.80429	7.015018
0.003	91.64916	1.415796	94.32774	3.575545	0.003	84.02149	5.847717
0.001	99.29097	3.554269	96.71744	2.475925	0.001	99.94344	4.854599
GI50	0.058	0.009	0.097	0.010	GI50	0.085	0.033
GI50 (μM)	0.075	0.012	0.150	0.015		0.095	0.037

	9			Adriamycin	
Conc.(ug/	Mean	SD	Conc.(ug/	Mean	SD
30	-7.64286	7.392413	3	-9.76562	4.974975
10	18.78572	5.051139	1	-2.42745	1.883871
3	45.19048	6.227485	0.3	18.7221	6.31633
1	84.40477	4.942222	0.1	43.24776	5.659775
0.3	99.69048	2.983827	0.03	95.70313	5.917283
GI50	2.840	0.257	GI50	0.088	0.006
GI50 (μM)	3.886	0.351	GI50 (μM)	0.162	0.012

MDA-MB-231

	1		2			3	
Conc.(ug/	Mean	SD	Mean	SD	Conc.(ug/	Mean	SD
0.3	10.15993	6.85252	14.2286	5.395022	0.3	22.7	5.468089
0.1	24.05927	9.824366	39.81656	5.522022	0.1	53.325	7.632987
0.03	47.48354	3.082368	56.13828	6.844498	0.03	69.025	3.191004
0.01	66.22765	8.548091	82.40827	7.277575	0.01	78.9	7.652016
0.003	88.31138	2.01527	90.68674	3.769985	0.003	85.35001	6.458327
0.001	95.46331	5.625924	97.29538	5.172824	0.001	94.37501	4.189175
GI50	0.026	0.007	0.050	0.011	GI50	0.083	0.005
GI50 (μM)	0.033	0.008	0.077	0.017		0.093	0.005

	9			Adriamycin	
Conc.(ug/	Mean	SD	Conc.(ug/	Mean	SD
30	-5.79678	4.827949	3	-9.70017	2.128615
10	10.67592	8.715566	1	-2.60141	2.926928
3	41.69651	8.581812	0.3	19.00353	3.598992
1	85.56401	4.961668	0.1	33.06878	3.821508
0.3	93.41988	4.140494	0.03	95.83333	3.181432
GI50	2.558	0.345	GI50	0.088	0.000
GI50 (μM)	3.500	0.472	GI50 (μM)	0.162	0.000

NCI-H23

	1			2	
Conc.(ug/	Mean	SD		Mean	SD
0.3	-3.49076	3.352121		-1.74538	3.861606
0.1	19.63552	6.464844		23.40862	8.618932
0.03	41.52977	6.339229		53.54209	3.941308
0.01	74.15297	4.754211		80.98049	8.203439
0.003	93.99384	5.662351		85.70328	3.921537
0.001	98.40862	3.034684		96.35523	6.757748
GI50	0.024	0.002		0.032	0.002
GI50 (μM)	0.031	0.002		0.050	0.004

	3	
Conc.(ug/	Mean	SD
0.3	15.68248	7.520562
0.1	29.81607	3.821055
0.03	43.99806	4.158877
0.01	76.30688	2.369097
0.003	89.54501	3.811231
0.001	98.30591	4.947187
GI50	0.032	0.003
	0.036	0.002

	9	
Conc.(ug/	Mean	SD
30	-9.16928	1.100491
10	15.88297	6.510523
3	42.58098	6.874614
1	75.52247	7.250737
0.3	94.95821	6.155571
GI50	2.372	0.131
GI50 (μM)	3.245	0.179

	Adriamycin	
Conc.(ug/	Mean	SD
3	-9.82323	2.312778
1	-7.77778	7.987254
0.3	16.79293	5.268929
0.1	24.31818	5.163302
0.03	94.77273	5.616363
GI50	0.079	0.002
GI50 (μM)	0.145	0.003

NUGC-3

	1			2	
Conc.(ug/	Mean	SD		Mean	SD
0.3	-14.7101	4.21399		-8.38164	5.54322
0.1	-2.46377	9.354366		20.45894	6.961609
0.03	31.57005	4.75978		34.37198	9.969145
0.01	64.27536	8.124902		70.67632	6.027879
0.003	83.88889	3.133152		78.2367	4.472621
0.001	96.40096	3.743156		93.64733	4.89832
GI50	0.015	0.003		0.018	0.004
GI50 (μM)	0.019	0.003		0.028	0.006

	3	
Conc.(ug/	Mean	SD
0.3	8.878504	5.691144
0.1	27.67393	4.304547
0.03	36.16303	4.689692
0.01	78.60851	4.506474
0.003	93.19833	3.310485
0.001	98.4164	3.387623
GI50	0.027	0.005
	0.030	0.006

	9	
Conc.(ug/	Mean	SD
30	-12.2608	7.994306
10	8.213078	6.814851
3	37.68294	5.165755
1	79.42125	6.893185
0.3	96.84526	5.066818
GI50	2.220	0.033
GI50 (μM)	3.037	0.045

	Adriamycin	
Conc.(ug/	Mean	SD
3	-8.65854	7.715083
1	-3.21951	2.290779
0.3	15.85366	3.889818
0.1	31.41463	7.157279
0.03	95.82926	8.800481
GI50	0.082	0.007
GI50 (μM)	0.151	0.014

PC-3

	1			2	
Conc.(ug/	Mean	SD		Mean	SD
0.3	-9.89968	4.425304		19.45617	6.278265
0.1	11.72122	6.475636		43.13622	6.881086
0.03	40.5227	4.103688		66.28828	7.61096
0.01	62.98838	8.489204		83.52692	7.835526
0.003	84.63569	4.472194		92.05386	6.348323
0.001	95.16896	3.700787		95.85533	5.219206
GI50	0.017	0.005		0.066	0.002
GI50 (μM)	0.022	0.006		0.103	0.002

	3	
Conc.(ug/	Mean	SD
0.3	19.96327	4.980578
0.1	35.62435	5.902043
0.03	58.18468	9.523778
0.01	89.95279	5.264314
0.003	94.22876	2.756334
0.001	98.74082	0.950197
GI50	0.055	0.010
	0.062	0.012

	9	
Conc.(ug/	Mean	SD
30	-8.6245	6.146929
10	14.20683	5.486072
3	32.32932	3.013164
1	76.17972	8.245725
0.3	94.20181	6.391286
GI50	2.010	0.251
GI50 (μM)	2.750	0.344

	Adriamycin	
Conc.(ug/	Mean	SD
3	-8.85847	5.478373
1	-3.79649	5.334938
0.3	19.39566	7.777665
0.1	37.80991	6.168429
0.03	94.39566	4.105782
GI50	0.080	0.003
GI50 (μM)	0.148	0.005

Figure S38. Results of the cytotoxicity test for **1-3**, and **9**.
